# What Controls the Orientation of TADF Emitters?

**DOI:** 10.3389/fchem.2020.00750

**Published:** 2020-09-04

**Authors:** Bilal A. Naqvi, Markus Schmid, Ettore Crovini, Prakhar Sahay, Tassilo Naujoks, Francesco Rodella, Zhen Zhang, Peter Strohriegl, Stefan Bräse, Eli Zysman-Colman, Wolfgang Brütting

**Affiliations:** ^1^Institute of Physics, University of Augsburg, Augsburg, Germany; ^2^Organic Semiconductor Centre, EaStCHEM School of Chemistry, University of St Andrews, St Andrews, United Kingdom; ^3^Macromolecular Chemistry, University of Bayreuth, Bayreuth, Germany; ^4^Institute of Organic Chemistry, Karlsruhe Institute of Technology, Karlsruhe, Germany; ^5^Institute of Biological and Chemical Systems – Functional Molecular Systems, Karlsruhe Institute of Technology, Eggenstein-Leopoldshafen, Germany

**Keywords:** OLEDs, TADF, emitter orientation, molecular orientation, emitter-host interaction

## Abstract

Thermally-activated delayed fluorescence (TADF) emitters—just like phosphorescent ones—can in principle allow for 100% internal quantum efficiency of organic light-emitting diodes (OLEDs), because the initially formed electron-hole pairs in the non-emissive triplet state can be efficiently converted into emissive singlets by reverse intersystem crossing. However, as compared to phosphorescent emitter complexes with their bulky—often close to spherical—molecular structures, TADF emitters offer the advantage to align them such that their optical transition dipole moments (TDMs) lie preferentially in the film plane. In this report, we address the question which factors control the orientation of TADF emitters. Specifically, we discuss how guest-host interactions may be used to influence this parameter and propose an interplay of different factors being responsible. We infer that emitter orientation is mainly governed by the molecular shape of the TADF molecule itself and by the physical properties of the host—foremost, its glass transition temperature T_g_ and its tendency for alignment being expressed, e.g., as birefringence or the formation of a giant surface potential of the host. Electrostatic dipole-dipole interactions between host and emitter are not found to play an important role.

## Introduction

Organic light-emitting diodes (OLEDs) are thin-film structures where photons are produced from radiative recombination of electron-hole pairs through an excited state of a molecular emitter material that is commonly embedded in a suitable host matrix to avoid aggregation and, thus, luminescence quenching (Tang et al., 1989). While the primary steps of exciton formation and decay are quantum mechanical in nature and also involve selection rules related to the spin of the involved species, the propagation and extraction of the produced radiation can be treated in a semi-classical dipole model (Barnes, 1998; Penninck et al., 2011). The external quantum efficiency η_ext_ of an OLED, i.e., the ratio between extracted photons from a device divided by the number of injected charges, is therefore split into an internal factor η_int_ comprising charge balance γ, spin statistics η_r_ and radiative exciton decay q_eff_, and an outcoupling factor η_out_ for the fraction of light that is actually emitted from the OLED and is visible to an observer (Tsutsui et al., 1997). Note that this separation is not strictly valid, because the radiative quantum efficiency is influenced by the device stack as well through the so-called Purcell effect, yielding an effective value q_eff_ (Nowy et al., 2008; Brütting et al., 2013).

$$\begin{array}{l} \eta_{ext}=\gamma \cdot \eta_{r}\cdot q_{eff}\cdot \eta_{out}\equiv \eta_{int}\cdot \eta_{out} \end{array}$$

**Figure 1:**
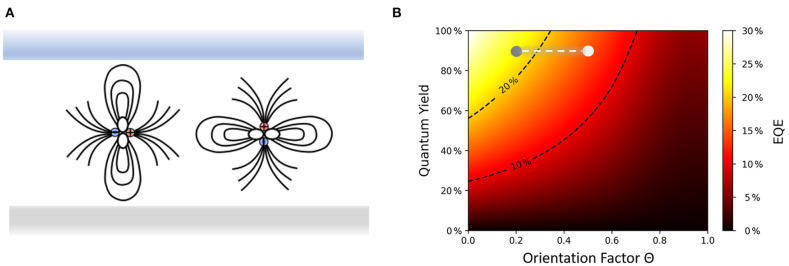
**(A)** The process of light emission in an OLED can be described as an oscillating dipole inside a micro-cavity formed by the two electrodes, which emits perpendicularly to its axis where ϑ is the angle between TDM vector and substrate normal (Schmidt et al., 2017). Light emitted from horizontally oriented dipoles (left) will be coupled out directly or goes to substrate modes, while vertical dipoles (right) couple mainly to surface plasmons and waveguide modes. Thus, to maximize the light outcoupling to air horizontally oriented TDMs are crucial. **(B)** Optical simulation that shows the dependency of η_ext_ on the orientation factor Θ (Θ = 0 for horizontally aligned TDMs and 1 for completely vertically aligned TDMs). The stack used for this simulation with DMAC-TRZ as the emitter is shown in the Supporting Material. The Θ value of DMAC-TRZ varies between 0.21 in an mCBP-CN matrix, which corresponds to 79% of the TDMs being horizontally aligned, and 0.52 in mCP, i.e., <50% horizontal TDMs in this case. The corresponding η_ext_ of the device is around 22% for the former case (gray point) and goes down to below 14% in the latter case (white point).

Thermally-activated delayed fluorescence (TADF) emitters—just like phosphorescent ones—can in principle harvest for 100% radiative excitons (η_r_ = 1) because the initially formed electron-hole pairs in the non-emissive triplet state can be efficiently converted into emissive singlets by reverse intersystem crossing (Uoyama et al., 2012). However, as compared to commercial iridium(III)-based phosphorescent complexes with their often close to spherical molecular structures, TADF emitters often possess similar shape to the host matrix molecules and offer the advantage to be aligned such that their optical transition dipole moments (TDMs) lie preferentially in the film plane (Figure 1A). Using the above mentioned semi-classical dipole model, it follows that the external quantum efficiency of a TADF OLED can be dramatically enhanced, if instead of an ensemble of randomly oriented emitter molecules, horizontally aligned TDMs prevail in the system (Figure 1B).

As described in detail in the Supporting Material, we use an order parameter Θ to quantify the degree of horizontal orientation of the emitting TDM (Schmidt et al., 2017), which is defined as the fraction of optical power emitted by vertical dipoles within the system. This parameter is equivalent to the second Legendre polynomial $P_{2}\left(\vartheta \right)=\;<\cos^{2}\vartheta >$, where ϑ is the angle between the substrate normal and the direction of the TDM vector (see Figure 1A). Ideally, the Θ values should be close to zero because the radiation from vertical dipoles remains trapped as wave-guided or surface plasmon modes and is not coupled out from an OLED.

This fact being known already for some time, the detailed mechanism driving non-isotropic orientation of molecular emitter materials in a guest-host system remained elusive. In an early work, Yokoyama has pointed out—at that time working with fluorescent-only materials—that the shape anisotropy of the molecules plays a decisive role (Yokoyama, 2011). The more rod-like (or disc-like) they are, the stronger their tendency to form optically anisotropic thin films, which he defined as the ratio of birefringence probed by ellipsometry. Alternatively, radiation pattern analysis under photoluminescence excitation (ADPL, “angular dependent photoluminescence”; see Supporting Material for details) was developed as a powerful method to study emitter orientation (Frischeisen et al., 2010). This technique enabled investigations on guest-host systems with only a small fraction of the light-emitting species embedded in a wider-gap host matrix. Surprisingly enough, non-isotropic radiation patterns indicating horizontal emitter orientation were observed even for systems where the host material alone does not show any anisotropy (Flämmich et al., 2011; Frischeisen et al., 2011).

An important step toward controlling emitter orientation came from the field of glass physics, where it was demonstrated that evaporated neat films of organic semiconductors can form anisotropic molecular glasses with their orientation being controlled by the temperature of the substrate T_S_ (and the evaporation rate) in relation to the glass transition temperature T_g_ of the organic material (Dalal et al., 2013). Specifically, it was shown that horizontal orientation of neat organic films leading to birefringence can be obtained for T_S_/T_g_ ≤ 0.8. Instead of varying the substrate temperature T_S_, Mayr showed that the same effect can be achieved in a guest-host system if hosts with different T_g_'s are used (Mayr and Brütting, 2015). Subsequently, TADF emitters with completely horizontal orientation could be achieved by film growth on cooled substrates and their positive effect for OLED efficiency was clearly demonstrated (Komino et al., 2016).

The current understanding of orientation in OLEDs has progressed substantially, in particular as it pertains to fluorescent as well as phosphorescent emitters (Schmidt et al., 2017; Kim and Kim, 2018). Spherically octahedrally coordinated phosphorescent Iridium(III) complexes can show some degree of horizontal orientation (typically <80%), but other reports suggest that this effect is even more pronounced for TADF emitters with up to 100% of the emitters being horizontally aligned (Byeon et al., 2018). Since thermal evaporation is a non-equilibrium process, molecular orientation in such non-crystalline materials is determined at the surface of the growing film (Jurow et al., 2016; Friederich et al., 2017; Kim and Kim, 2018). Depending on substrate temperature, evaporation rate, molecular shape, and other—perhaps yet unknown—factors, the molecules at the surface may or may not have enough time to diffuse around, reorient and equilibrate with the underlying film, before they are covered by the next deposited layer such that their orientation becomes frozen (Ediger et al., 2019). Thus, film growth and morphology are kinetically controlled processes.

**Figure 2:**
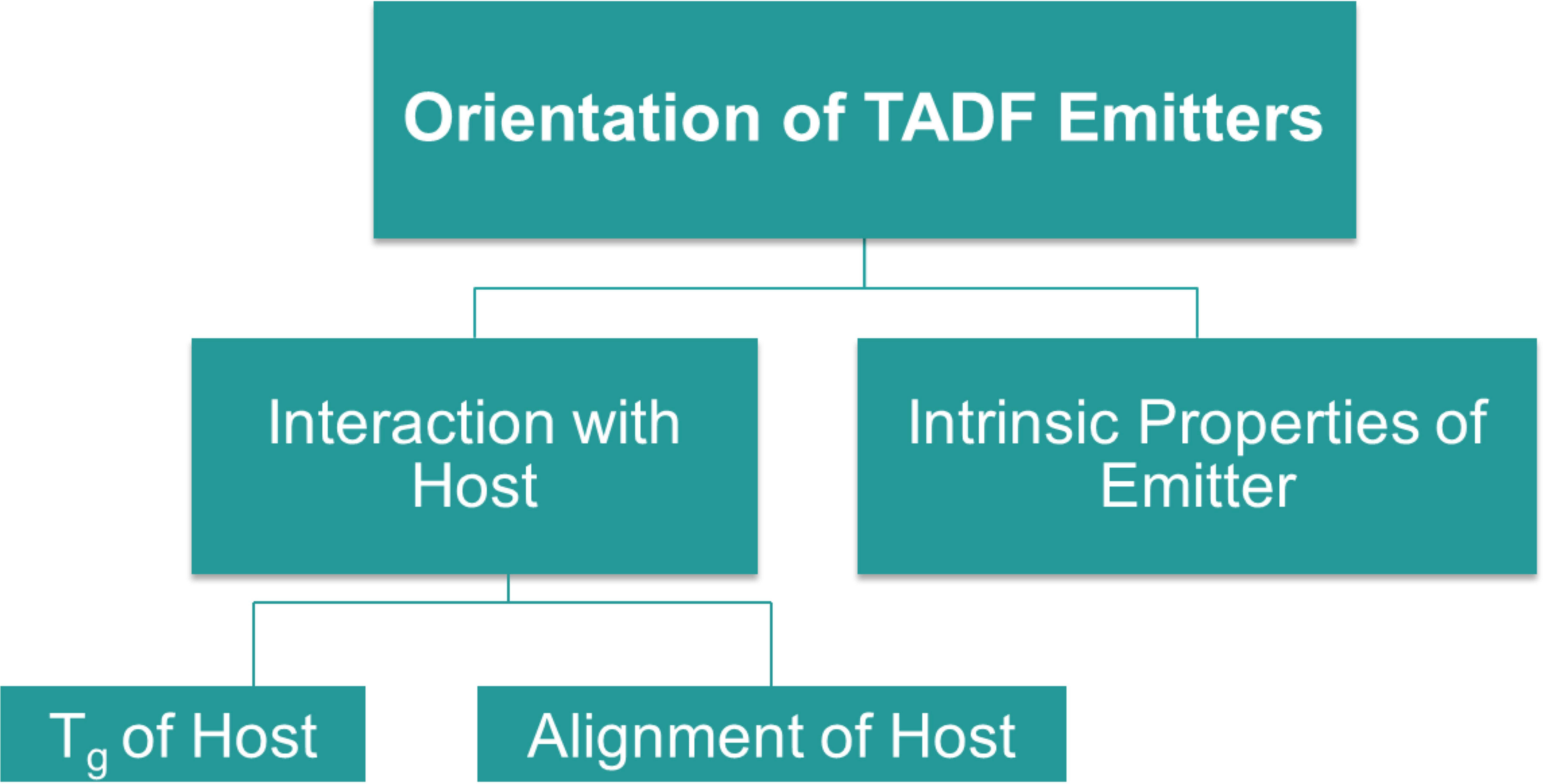
Proposed model for the dominant factors affecting TADF emitter orientation. (See text for further details).

In this article, we address the question of which factors control the orientation of TADF emitters. Specifically, we discuss if it is an intrinsic property of the emitter and how guest-host interactions may be used to influence this parameter. Ultimately, we propose an interplay of different factors being responsible, as shown schematically in Figure 2.

## Basic Approach

OLEDs based on TADF emitters can yield up to 100% internal quantum efficiency because all the generated triplet excitons under electrical operation are ideally converted to singlets through fast reverse intersystem crossing (Adachi, 2014). In order for this to happen the energy difference, ΔE_ST_, between singlet and triplet excited states must be sufficiently small (of the order of few k_B_T only) to be thermally enabled (Wong and Zysman-Colman, 2017). As ΔE_ST_ is governed by the exchange integral of the frontier orbitals responsible for the transition to these excited states, which is usually defined as the wavefunction overlap of HOMO and LUMO, i.e., the respective highest occupied and lowest unoccupied molecular orbitals (Penfold, 2015; Yersin, 2018)

$$\begin{array}{l} \;\Delta E_{ST}\propto \;<{\text{Ψ}}_{HOMO}\left|\frac{1}{r_{12}}\right|{\text{Ψ}}_{LUMO}> \end{array}$$

the key is to separate them spatially, which is typically done by using a twisted donor-acceptor molecular architecture that strongly electronically decouples these two moieties. However, because the HOMO-LUMO overlap also is proportional to the oscillator strength for the radiative decay to the ground state, it should not become too small lest efficient luminescence will no longer be possible (Weissenseel et al., 2019).

Accordingly, the optical transition dipole moment, which is given by a similar expression,

$$\begin{array}{l} {\vec{p}}_{TDM}\propto \;<{\text{Ψ}}_{HOMO}\left|{\vec{r}}_{12}\right|{\text{Ψ}}_{LUMO}> \end{array}$$

also depends on the distribution of these orbitals on the molecule. TADF compounds thus typically possess emissive charge-transfer (CT) states and the direction of their TDM often coincides with (or is very close to) the long molecular axis between donor and acceptor units. Note that there are some exceptions like the well-known 4CzIPN, which is an almost spherical molecule (Hasegawa et al., 2018). Nevertheless, the inline alignment of TDM and long molecular axis of TADF emitters holds great potential for manipulating their TDM orientation, and thus the light outcoupling from the OLED, by controlling molecular orientation upon thermal evaporation of films.

## Materials and Methods

**Figure 3:**
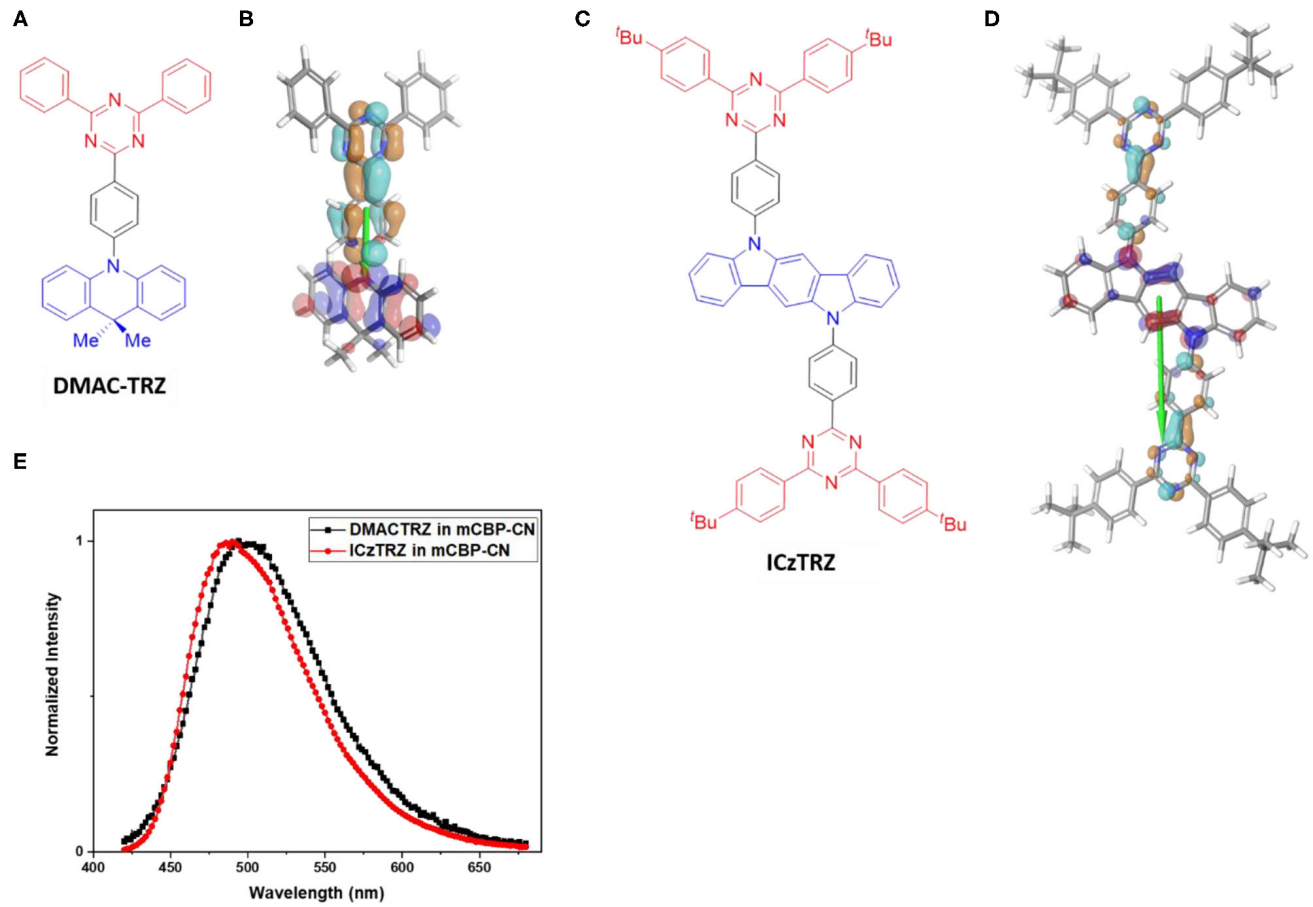
**(A,C)** Schematic structures of the two TADF emitters DMAC-TRZ and ICzTRZ with the respective donor part colored blue and the acceptor red. **(B,D)** Calculated HOMO (blue/red) and LUMO (cyan/brown) electron densities and the resulting TDM vectors (green). **(E)** Photoluminescence spectra of thin films with 10% each of DMAC-TRZ (black) and ICzTRZ (red) doped in a mCBP-CN host matrix.

Figure 3 shows the two emitter materials, DMAC-TRZ and ICzTRZ, used in this study together with their photoluminescence spectra. Both molecules are TADF emitters and emit sky-blue light. Its constituents, DMAC as donor and TRZ as acceptor, are frequently used in other TADF emitters as well and can, thus, be considered as prototypical building blocks (Wong and Zysman-Colman, 2017). ICzTRZ is a newly synthesized TADF emitter (Zhang et al., 2020), having the said TRZ as one compartment, which has almost identical emission spectrum to DMAC-TRZ (see Figure 3E) and, thus, similar energetics. Also shown in that figure are the calculated electron density distributions of the HOMO and LUMO (details in the Supporting Material). It is evident that HOMO and LUMO are spatially separated in both emitters. In DMAC-TRZ, the HOMO is located at the acridine donor and the LUMO at the triazine acceptor, while in ICzTRZ the HOMO resides at the indocarbazole donor whereas the LUMO is extended on the two triazine acceptors on both sides of the central core. Remarkably, they are largely different in both size and permanent dipole moment (PDM): DMAC-TRZ consists of a single DA building block, while ICzTRZ follows a symmetric ADA design and is therefore roughly twice as long. Moreover, because of their different designs, the DA-type system DMAC-TRZ is a polar molecule (2.01 D) while the ADA-type molecule ICzTRZ is not (0.33 D). Likewise, the longer and heavier ICzTRZ possesses a higher glass transition temperature T_g_ of 253°C, whereas the shorter and lighter DMAC-TRZ has a comparatively lower T_g_ of 93°C. The calculated HOMO and LUMO distributions shown in Figure 3 support this notion and, particularly, indicate that the TDM is parallel to the long molecular axis.

**Figure 4:**
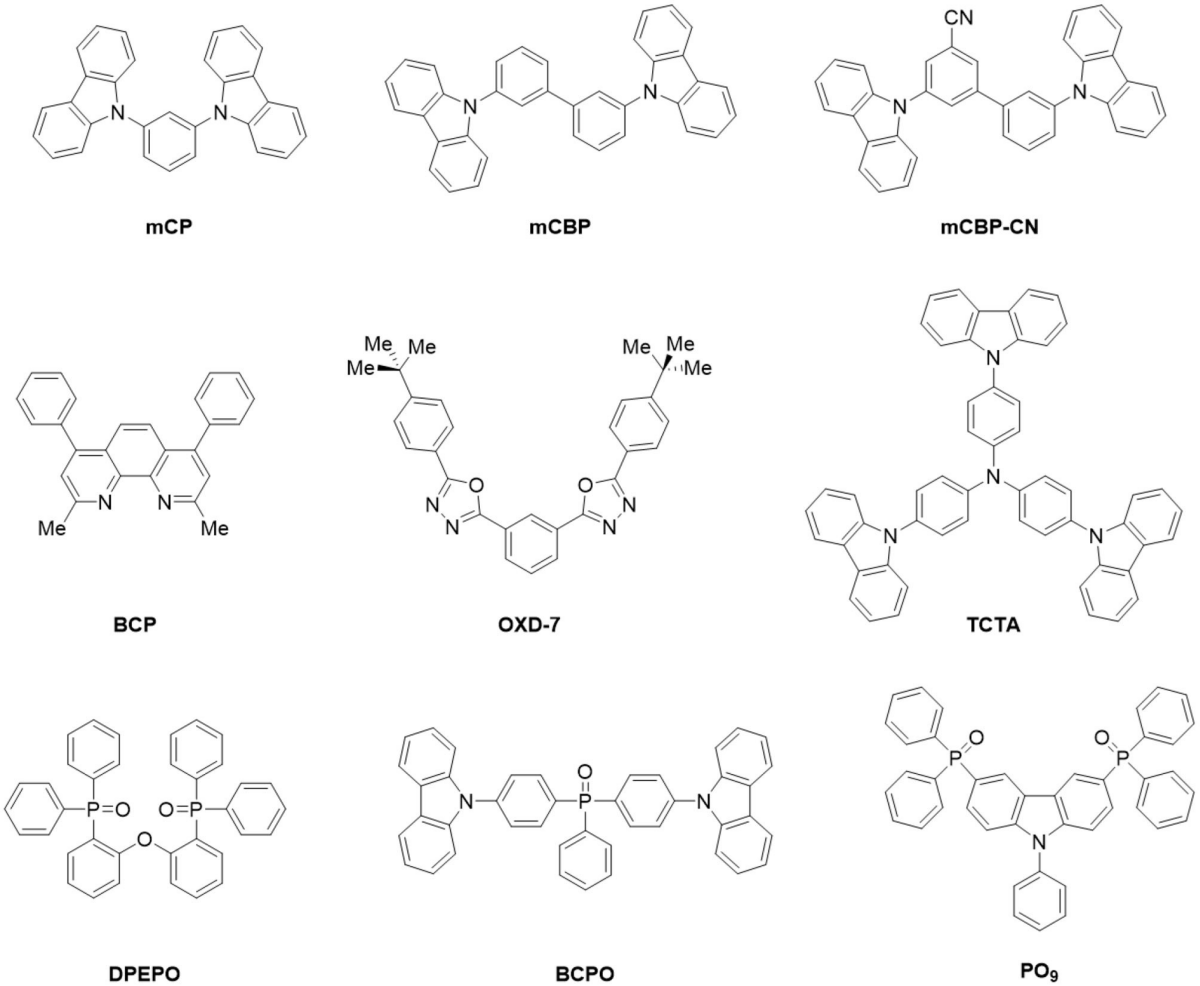
Schematic structures of hosts used in this study.

These two TADF emitters were co-evaporated with up to nine different host materials (Figure 4). In general, a suitable host material should have a larger optical gap to allow for energy transfer to and emission from the guest molecules; however, not all of these hosts are actually suitable hosts for efficient OLEDs because of additional requirements with respect to their triplet levels and their charge transport properties. Nevertheless, these host materials were chosen because they cover a wide range of glass transition temperatures T_g_ (see Table 1), which is expected to have an effect on orientation as discussed above. Moreover, since a CT excitation results in a polar state, TADF emitters are known to be strongly affected by the polarity of the surrounding host material (Dos Santos et al., 2016). Thus, almost all of the chosen hosts also have non-negligible permanent electric dipole moment as specified in Table 1. This also allows investigating the potential influence of electrostatic dipole-dipole interactions between host and emitter on the orientation process of the latter. Furthermore, these host matrices do not just consist of randomly oriented polar species but, upon thermal evaporation, some of them form films with a macroscopic dielectric polarization, which can be equivalently expressed as a non-vanishing surface charge and is often termed the giant surface potential (GSP). These values can be measured by Kelvin probe or impedance spectroscopy, as discussed in the Supporting Material, and are also given in Table 1. Finally, the Λ parameter quantifies alignment of the host PDMs as will be discussed later in detail.

**Table 1:** Physical properties of host materials used in this study.

**Host**	**T_**g**_ (°C)**	**PDM (D)**	**GSP (mV/nm)**	**Degree of PDM alignment Λ**
BCP	62	2.8	33	0.050
mCP	65	1.35	−3.9	0.015
OXD-7	77	5.5	68	0.069
mCBP	92	1.57	0	0
DPEPO	93	5.5	61.7	0.071
mCBP-CN	113	3.7	62.5	0.11
BCPO	137	3.5	163	0.33
PO_9_	122	6.7	45.6	0.05
TCTA	151	0	0	0

*PDMs were calculated using Schrodinger Maestro Software Package. GSP was measured using impedance spectroscopy and from the measured GSP a Λ parameter was calculated quantifying the degree of the hosts' PDM alignment as discussed in detail in the Supporting Material. The hosts' glass transition temperatures T_g_ were either taken from literature, as indicated, or measured by differential scanning calorimetry in this study, as described in the Supporting Material. ^a^Mayr and Brütting (2015), ^b^Chou and Cheng (2010), and ^c^Measured values using DSC–details in Supporting Material*.

## Results and Discussion

**Figure 5:**
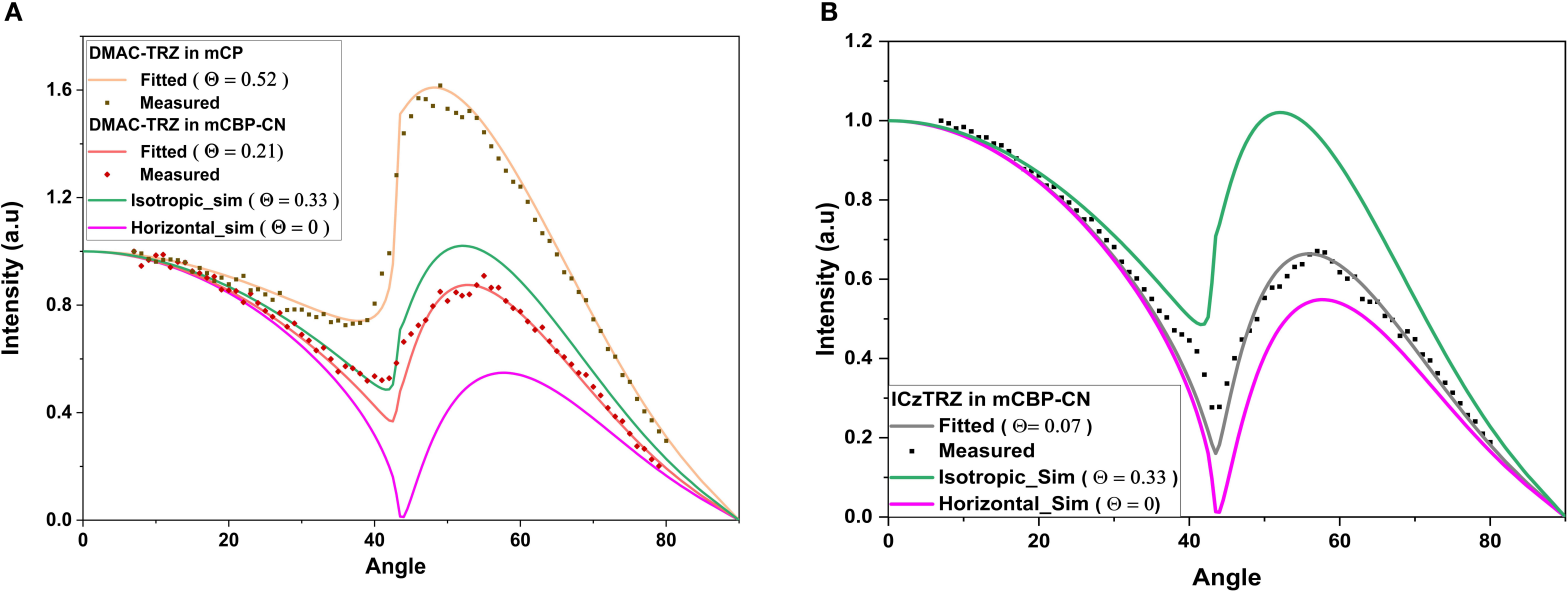
**(A)** Measured and fitted angular dependent photoluminescence for 10 wt% DMAC-TRZ in mCP (Θ = 0.52) and mCBP-CN (Θ = 0.21). **(B)** Measured and fitted ADPL for 10 wt% ICzTRZ in mCBP-CN. Simulated curves for complete horizontal orientation (Θ = 0, in pink) and isotropic (Θ = 0.33, in green) are also shown for comparison.

DMAC-TRZ and ICzTRZ were co-evaporated with the different hosts as thin films on glass substrates. These films were then subjected to ADPL measurements and numerical simulation (details can be found in the Supporting Material). To avoid artifacts caused by crystallization of the host matrix (especially, for low-T_g_ materials), samples were measured as soon as possible after film deposition, but in any case, on the same day of their fabrication. Figure 5 shows exemplary results for the two emitters together with fits to determine the emitter orientation as well as simulations indicating the expected shape of the curves for the limiting cases of Θ = 0.0 (completely horizontal) and Θ = 0.33 (isotropic). The determined orientation parameters of the TDMs of both emitters in the different hosts are summarized in Table 2.

**Table 2:** Determined orientation factors (Θ) for 10 wt% doping ratio of DMAC-TRZ and ICzTRZ in different hosts.

**Host**	**TDM orientation factor Θ**	**TDM orientation factor**
	**for DMAC-TRZ**	**Θ for ICzTRZ**
mCP	0.52 ± 0.01	0.12 ± 0.01
mCBP	0.48 ± 0.01	0.09 ± 0.02
BCP	0.42 ± 0.03	-
DPEPO	0.36 ± 0.02	0.06 ± 0.02
OXD-7	0.33 ± 0.01	-
PO_9_	0.27 ± 0.01	-
BCPO	0.24 ± 0.01	-
TCTA	0.24 ± 0.02	0.07 ± 0.03
mCBP-CN	0.21 ± 0.02	0.07 ± 0.02

*See Supporting Material for a complete set of the measured and fitted ADPL data*.

It was observed that ICzTRZ is stronger horizontally oriented in five of these hosts as compared to DMAC-TRZ. The orientation factor for DMAC-TRZ ranges from Θ = 0.52 in mCP to Θ = 0.21 in an mCBP-CN matrix, while for ICzTRZ the values are not as divergent. ICzTRZ has the highest orientation factor of Θ = 0.12 in mCP and the lowest is Θ = 0.06 in DPEPO, which is among the best values reported for TADF emitters (Mayr et al., 2014; Byeon et al., 2018; Tanaka et al., 2020). It is apparent from this analysis that the TDM orientation in DMAC-TRZ is affected much more strongly by the nature and polarity of the host material than in ICzTRZ.

As mentioned in the introduction, Yokoyama et al. found a correlation between anisotropic molecular shape and the tendency for horizontal molecular orientation for a series of rod-like fluorescent dyes (Yokoyama, 2011). This effect is also seen in the case of these two TADF emitter molecules, where the TDM is almost parallel to the long molecular axis. Therefore, the long ICzTRZ molecule has a much stronger horizontal TDM orientation as compared to the short DMAC-TRZ molecule. Moreover, the anisotropy factor of ICzTRZ is less effected by different hosts because alignment is predominantly induced by the extended molecular shape of the emitter.

We recall that the different TDM orientations have important consequences for light-outcoupling in OLEDs. As already shown in Figure 1B, the simulated EQE of DMAC-TRZ in an OLED stack is expected to vary from <14% in mCP as the host to about 22% if the orientation obtained in mCBP-CN is taken (see the Supporting Material for actual device data of DMAC-TRZ). OLEDs with ICzTRZ have been the subject of a separate study (Zhang et al., 2020) where an EQE_max_ of 22.1% has been achieved in mCBP as the host.

**Figure 6:**
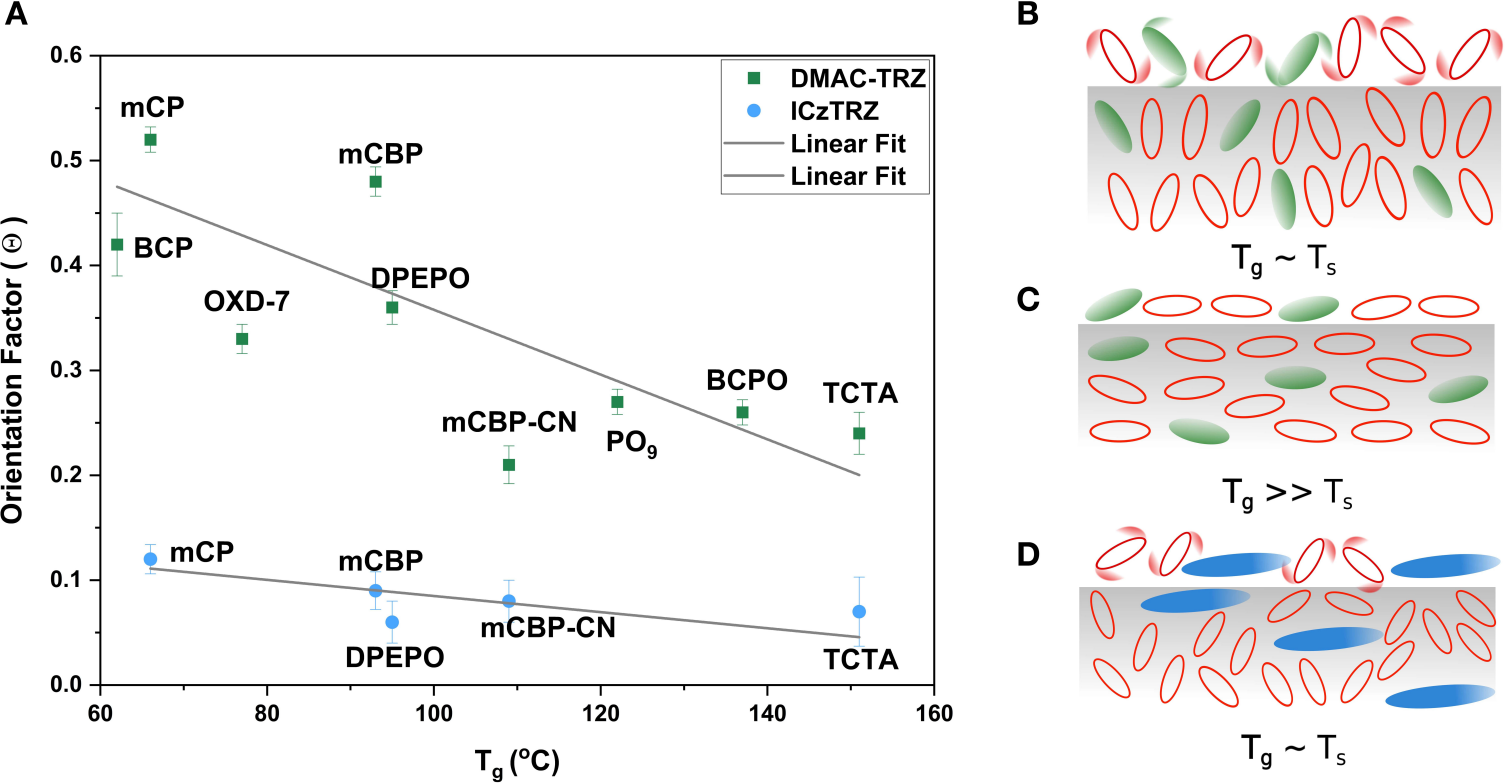
**(A)** Dependency of the emitter's TDM orientation factor (Θ) vs. T_g_ of the hosts. **(B–D)** Schematic illustration of the effect of T_g_ (mobility of host molecules) on the orientation of emitter molecules. Host molecules are shown in red, DMAC-TRZ in green, and ICzTRZ in blue. **(B)** Enhanced molecular mobility and adoption of vertical orientation of host and DMAC-TRZ molecules for T_g_ ~ T_S_, while in **(C)** pronounced horizontal orientation is observed when T_g_ >> T_S_. **(D)** The long ICzTRZ is relatively unaffected by the mobile host molecules regardless of their T_g_'s.

We now turn to the question of how the large variation of orientation in DMAC-TRZ can be further understood. Apparently, the molecule is not long enough to be intrinsically oriented horizontally. Thus, guest-host interactions become the dominant factor. Following the surface equilibration model discussed in the introduction, we therefore plot the extracted Θ values vs. the T_g_'s of the host in Figure 6A. As predicted by this model, the orientation depends strongly on the host: the larger its T_g_, the lower are the Θ values. Since the substrate temperature is always kept at or slightly above room temperature (T_S_ ~ 300K), we arrive at a ratio of T_S_/T_g_ ~ 0.9 for low-T_g_ hosts like mCP or BCP, while the highest T_g_ hosts yield a ratio of about 0.7. According to the work of Ediger et al. these values are above and below the critical value (T_S_/T_g_ ~ 0.85), respectively, to change molecular orientation from more vertical in the former case, to more horizontal in the latter (Ediger et al., 2019).

Thus, we propose a possible model for this behavior based upon these trends and previous studies (Mayr and Brütting, 2015; Ediger et al., 2019). If the substrate temperature is close to the T_g_ of the host material, molecules landing on the surface will have high mobility and, consequently, enough time to adapt to the surface equilibrium structure, which consists of predominantly vertically aligned molecules (Figure 6B). However, if T_g_ >> T_S_, the molecules will not have enough time to diffuse and reorient on the surface before being immobilized by the next layer of molecules and will thus not be able to equilibrate (Figure 6C). Thus, the initial horizontal orientation at the surface will be effectively frozen because mobilities in the bulk are orders of magnitude lower than at the surface of the film (Ediger et al., 2019). If the substrate temperature is fixed and we co-deposit an emitter with hosts having different T_g_, a similar effect will therefore be observed. This effect is seen when DMAC-TRZ is co-deposited with hosts with a range of different T_g_. It is highly vertical (Θ = 0.52) in mCP, which has T_g_ of 62°C, while this factor becomes less than half as large (Θ = 0.24) in a host with high T_g_ like BCPO and TCTA.

This trend was also followed for ICzTRZ but, overall, it shows horizontal orientation in all the different hosts. The highest value of the orientation factor (Θ = 0.12) for ICzTRZ is much lower than the lowest in DMAC-TRZ (Θ = 0.21). Thus, we believe that ICzTRZ is not much affected by the mobility of host molecules owing to its long molecular shape and its high T_g_ as neat material (Figure 6D). This is confirmed by the fact that the strongest horizontal orientation is already observed in DPEPO as host, which has a moderate T_g_, and does not further improve even for the highest T_g_ host TCTA.

However, there are also some outliers for DMAC-TRZ in Figure 6A. This means that for some hosts we see a stronger horizontal alignment than expected from the T_g_ alone; see e.g., BCP in comparison to mCP, DPEPO compared to mCBP, or OXD-7 and mCBP-CN. All of them are relatively polar materials. Thus, we have to extend the above presented model to include polarity of the host, but more importantly, the possibility of orientational order of the host itself.

To this end, the concept of spontaneous orientation polarization is very useful. It is known that many polar organic semiconductors exhibit a giant surface potential in thin films grown by vacuum deposition. In this case, the orientation of their permanent electrical dipole moments has a preferential alignment perpendicular to the film plane, i.e., in the vertical direction (Noguchi et al., 2019). This can even occur if the molecules have almost spherical structure, like Alq_3_, and do not exhibit any optical anisotropy. The GSP of the host materials has been determined by Kelvin probe and impedance spectroscopy as discussed in the Supporting Material. The obtained values are listed in Table 1.

**Figure 7:**
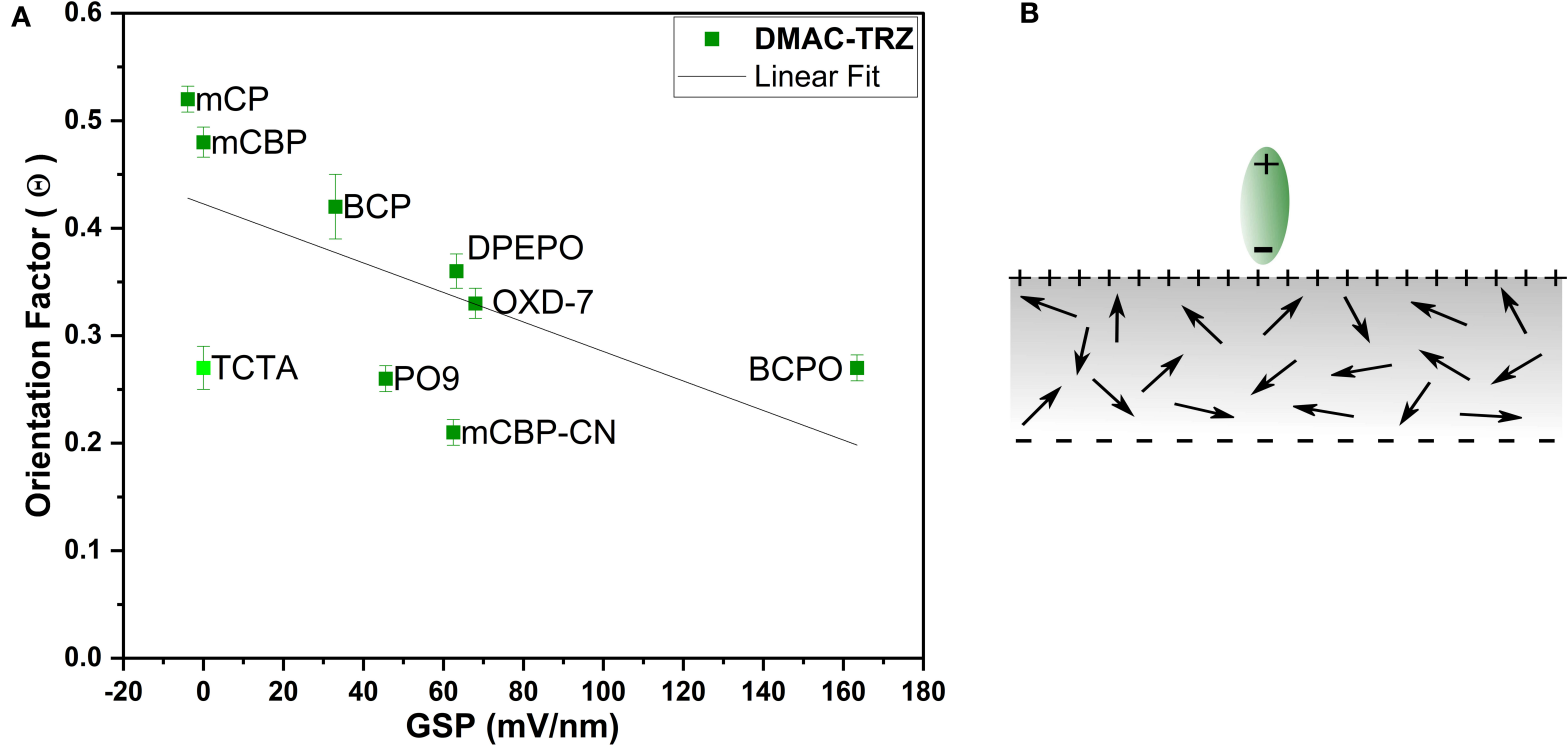
**(A)** Dependency of the emitter's TDM orientation factor (Θ) vs. the GSP of the hosts with a linear fit. **(B)** Schematic illustration of an organic film with GSP and electrostatic interaction between an oncoming emitter molecule (green) and the surface charge leading to vertical alignment of the emitter molecule.

Figure 7A shows the TDM orientation parameter Θ of DMAC-TRZ plotted vs. the GSP of the host materials. Apparently, there seems to be a trend of more horizontal TDM orientation with increasing surface potential; but still some data points clearly deviate from the fitted linear trendline. First and most obviously, this is TCTA, which does not have a GSP, because it is a non-polar molecule. Secondly, also BCPO and mCBP-CN show stronger orientation as compared to host materials with similar GSP. This raises the question, if the GSP, i.e., the density of polarization charges sitting at the surface of the film is a suitable parameter to explain TADF emitter orientation. In a simple electrostatic interaction model (see Figure 7B) the electric field originating from the positive surface charge would rather align the arriving polar TADF emitter molecules in the vertical direction. The larger the GSP, the more vertical alignment would be expected. This is obviously not observed, because for DMAC-TRZ the PDM is roughly parallel to the TDM, and the latter is more and more horizontal the larger the GSP of the host becomes. Thus, the argument using the macroscopic orientation polarization of the host materials has to be revised.

In general, the degree of alignment in such host materials is rather low. If an order parameter Λ is defined as the ratio between the measured GSP and the theoretically possible value for perfect vertical alignment of its PDMs (see Supporting Material for details), one typically finds numbers in the range 5–10% (Jäger et al., 2016). This means that either most of their PDMs are randomly oriented with just a small net alignment in vertical direction or that most of the PDMs align pairwise antiparallel so that their net dipole moment vanishes.

**Figure 8:**
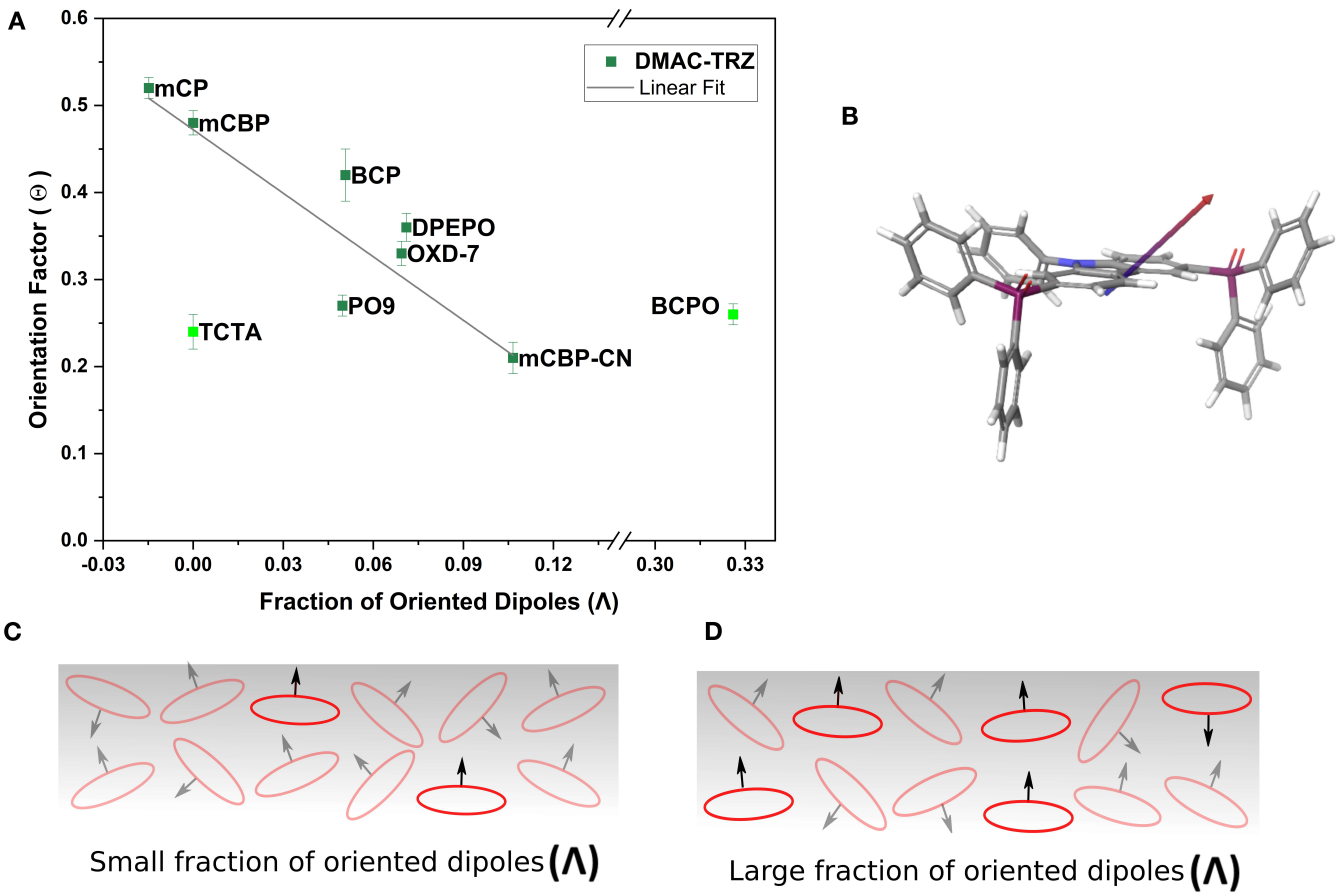
**(A)** Dependency of emitter's TDM orientation factor (Θ) vs. the fraction of oriented PDMs (Λ) of the hosts with a linear fit. **(B)** 3D structure of PO_9_ with its PDM pointing outwards of the basal plane of the carbazole core. **(C,D)** Schematic illustration of the fraction of oriented host molecules with a direction of permanent dipole moments pointing perpendicular to their long axis.

In order to investigate the potential influence of host alignment on TDM orientation we have analyzed the order parameter Λ for all the host materials (values were given already in Table 1). Figure 8A shows the TDM orientation Θ of the emitter DMAC-TRZ plotted vs. the PDM orientation parameter Λ of the host. In this case, the correlation is significantly improved compared to that in Figure 7A, which indicates that the horizontal alignment of the emitter molecules is not driven by an electrostatic interaction according to the GSP at the film surface, but rather by the higher degree of ordering of the host molecules.

At first glance this may appear somewhat counterintuitive, but one must be aware that many of the studied host molecules have a PDM pointing perpendicular to the long molecular axis (Figure 8B and Supporting Material) This is true specifically for phosphine oxides like DPEPO, BCPO, and PO_9_, but also to some extent for mCBP-CN and OXD-7. Thus, a higher degree of vertical alignment of their PDMs actually means that these host molecules preferentially lie flat on the film surface (see Figures 8C,D). We note that this consideration is true even for the remaining outlier in Figure 8A: TCTA. It does not have a PDM; therefore, Λ = 0. However, TCTA is known to be birefringent with the ordinary component of the refractive index being about 0.15 larger than the extraordinary one (see the optical constants in the Supporting Material), indicating that it adopts a preferential horizontal orientation. This is not surprising in view of its high T_g_ if the above discussed surface equilibration model is being considered not only for the emitter molecules but for the host as well.

Finally, we also want to note that chemical interactions between host and emitter molecules may cause specific combinations of them to orient better than predicted by the general T_g_ trend. It has been reported recently, that specific molecular units may allow π-π stacking or weak hydrogen bonds between host and emitter and, thus, promote horizontal orientation (Watanabe et al., 2019; Sasabe et al., 2020). Probably, this could contribute to the observation that DMAC-TRZ is relatively more horizontally aligned in mCBP-CN as compared to other hosts with similar physical properties.

## Conclusions

The answer to the question: “What controls the orientation of TADF emitters?” has—at least—three components. In the first place, it is an intrinsic property of the emitter molecule itself. The longer and more rod-like it is, the stronger will be its tendency to lie down when evaporated on a surface. In addition, due to their relatively high molecular mass, such emitter molecules will be affected only little by the arrangement of surrounding host molecules. In this respect, the ADA (or, equivalently, DAD) design principle of many TADF emitters can be regarded as highly beneficial for horizontal alignment.

In the second place, if the TADF emitter is shorter (containing only one DA building block), the host matrix takes over the dominant role for determining emitter orientation and, in particular, the glass transition temperature of the host is of paramount importance. A high T_g_ reduces the surface diffusivity of molecules such that they do not have enough time to equilibrate and, thus, often adopt the favored lying flat orientation. As a third factor, we could identify alignment of the host material itself as an additional parameter to promote TADF emitter orientation.

Although one can already find several examples in the literature, where researchers have used these guidelines on purpose (or perhaps even without knowing), we anticipate that designing TADF emitters in a way to promote their horizontal alignment could further boost this third generation of OLEDs to outcompete phosphorescent ones, specifically in the blue spectral region.

## Data Availability Statement

All datasets generated for this study are included in the article/Supplementary Material.

## Author Contributions

BN, MS, EC, EZ-C, and WB conceived the project. BN prepared samples and performed angular dependent PL measurements and their analysis to obtain TDM orientation. MS and EC performed TD-DFT on the emitter molecules. MS further calculated PDMs of host materials and analyzed their alignment by impedance spectroscopy. OLEDs were prepared and analyzed in experiment and simulation by PSa and TN. FR measured glass transition temperatures of the emitters and some of the hosts. ZZ synthesized the emitter ICzTRZ. PSt, SB, EZ-C, and WB supervised the project. All authors discussed the results and commented on the manuscript.

## Conflict of Interest

The authors declare that the research was conducted in the absence of any commercial or financial relationships that could be construed as a potential conflict of interest.
