# Corrigendum: What Controls the Orientation of TADF Emitters?

**DOI:** 10.3389/fchem.2021.632639

**Published:** 2021-02-17

**Authors:** Bilal A. Naqvi, Markus Schmid, Ettore Crovini, Prakhar Sahay, Tassilo Naujoks, Francesco Rodella, Zhen Zhang, Peter Strohriegl, Stefan Bräse, Eli Zysman-Colman, Wolfgang Brütting

**Affiliations:** ^1^ Institute of Physics, University of Augsburg, Augsburg, Germany; ^2^ Organic Semiconductor Centre, EaStCHEM School of Chemistry, University of St Andrews, St Andrews, United Kingdom; ^3^ Macromolecular Chemistry, University of Bayreuth, Bayreuth, Germany; ^4^ Institute of Organic Chemistry, Karlsruhe Institute of Technology, Karlsruhe, Germany; ^5^ Institute of Biological and Chemical Systems – Functional Molecular Systems, Karlsruhe Institute of Technology, Eggenstein-Leopoldshafen, Germany

**Keywords:** OLEDs, TADF, emitter orientation, molecular orientation, emitter-host interaction

In the original article, there was a mistake in Supplementary Figure S8 of the Supplementary Material, and derived from that, in Table 1 as well as Figures 7A, 8A as published. The measured values of the GSP for two of the host materials, viz. BCPO and PO_9_, were interchanged by mistake. These values also resulted in a wrong calculation of the degree of PDM alignment (Λ). The corrected Table 1 as well as Figures 7A, 8A are attached below.

**Figure 7:**
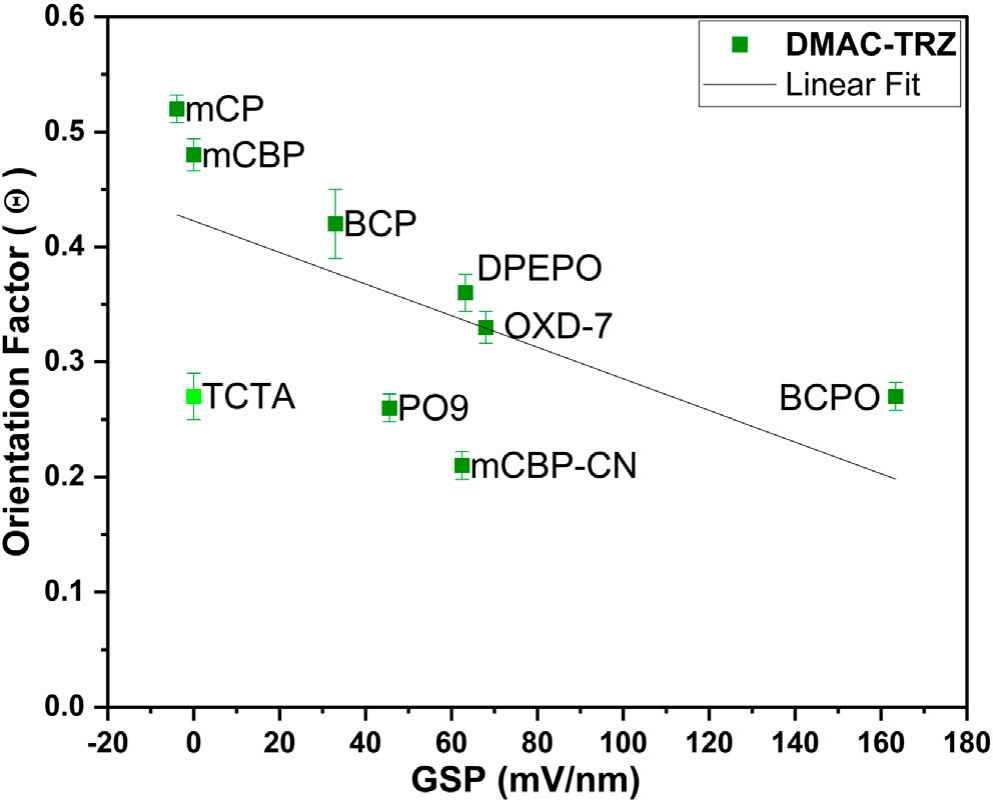
**(A)** Dependency of the emitter’s TDM orientation factor (Θ) vs. the GSP of the hosts with a linear fit.

**FIGURE 8:**
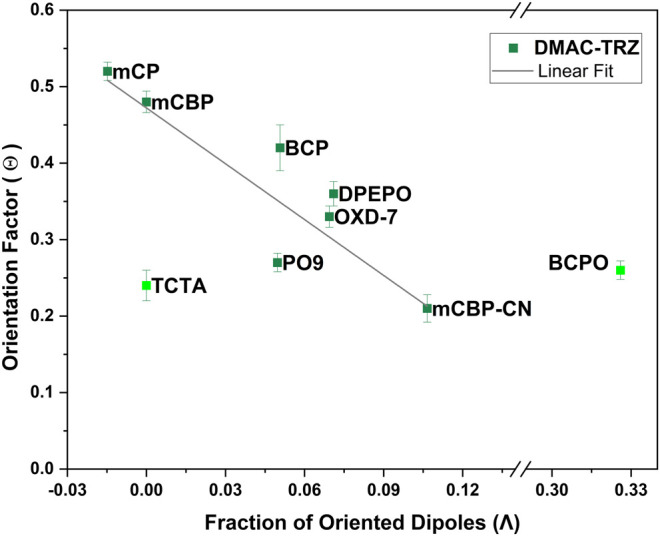
**(A)** Dependency of emitter’s TDM orientation factor (Θ) vs. the fraction of oriented PDMs (Λ) of the hosts with a linear fit.

**TABLE 1:** Physical properties of host materials used in this study.

Host	T_g_ (°C)	PDM (D)	GSP (mV/nm)	Degree of PDM alignment Λ
BCP	62	2.8	33	0.050
mCP	65	1.35	−3.9	0.015
OXD-7	77	5.5	68	0.069
mCBP	92	1.57	0	0
DPEPO	93	5.5	61.7	0.071
mCBP-CN	113	3.7	62.5	0.11
BCPO	137	3.5	163	0.33
PO_9_	122	6.7	45.6	0.05
TCTA	151	0	0	0

## Supplementary Material

The authors apologize for this error and state that this does not change the scientific conclusions of the article in any way. The original article and the Supporting Material have been updated.

