# Supplementary material for: What Controls the Orientation of TADF Emitters?
Source: Front Chem. 2020 Sep 4;8:750. doi: 10.3389/fchem.2020.00750 (PMC7500207; doi:10.3389/fchem.2020.00750)
Supplement: Supplementary file 1 [file Data_Sheet_1.pdf]

**What controls the orientation of TADF emitters? – Supplementary Material**

**Bilal A. Naqvi[1], Markus Schmid[1], Ettore Crovini[2], Prakhar Sahay[1], Tassilo Naujoks[1], Francesco Rodella[3], Zhen Zhang,[4] Peter Strohriegl[3], Stefan Bräse,[4,5] Eli Zysman-Colman[2], Wolfgang Brütting[1]***

[1] Institute of Physics, University of Augsburg, 86159 Augsburg, Germany

[2] Organic Semiconductor Centre, EaStCHEM School of Chemistry, University of St Andrews, St Andrews, Fife, KY16 9ST, United Kingdom

[3] Macromolecular Chemistry, University of Bayreuth, 95440 Bayreuth, Germany[5]

[4]Institute of Organic Chemistry, Karlsruhe Institute of Technology (KIT), Fritz-Haber-Weg 6, 76131 Karlsruhe, Germany

[5]Institute of Biological and Chemical Systems – Functional Molecular Systems (IBCS-FMS), Karlsruhe Institute of Technology (KIT), Hermann-von-Helmholtz-Platz, D-76344, Eggenstein-Leopoldshafen, Germany

*E-mail: bruetting@physik.uni-augsburg.de

**Contents:**

1. Angular dependent photoluminescence (ADPL) spectroscopy
2. Refractive indices of host materials
3. TD-DFT of emitter molecules
4. Permanent dipole moments (PDMs) of host molecules
5. Impedance spectroscopy and permanent dipole orientation of host materials
6. Device structure and characterisation
7. Differential scanning calorimetry on host materials
8. References

## 1. Angular dependent photoluminescence (ADPL) spectroscopy

Thin films of emitter and host were co-evaporated in high vacuum on a pre-cleaned quartz glass substrate. 10 % doping ratio of emitter molecules in the host matrix was obtained by controlling the deposition rate. This substrate was then glued with an index matching fluid on a fused-silica prism which was mounted on a rotating stage. The organic film was then optically excited by a UV laser (λ = 325 nm) under normal incidence and rotated from -90° to +90° as shown schematically in Fig. S1. The luminescence was recorded with a grating spectrograph coupled to a liquid-nitrogen cooled charge-coupled device ((Princeton Instruments Acton 2300i with PyLoN detector) in s and p polarization mode. Both signals were then subjected to numerical simulation to check the film thickness (s-pol.) and, most importantly, to obtain the orientation factor (Θ) from the p-pol. component:

$$\Theta = \frac{\Sigma p_z^2}{\Sigma p^2}$$

Here $\Sigma p_z^2$ is the sum of the power emitted by vertically oriented dipoles and $\Sigma p^2$ is the total power emitted by all emitting dipoles. The detailed method description can be found in references (Frischeisen et al., 2010) & (Schmidt et al., 2017).

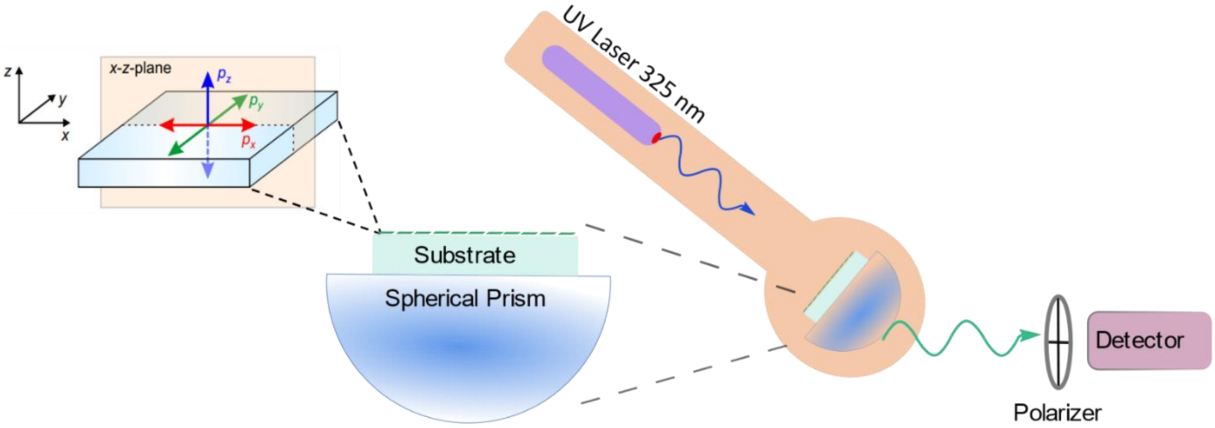


*Figure S1: Schematic illustration of the measurement setup used for determining the TDM orientation factor of emissive thin films.*

The input parameters for the numerical simulation are the film thickness and the optical parameters of the film, i.e. refractive index (n) and extinction coefficient (κ) in dependence on wavelength, which were determined beforehand by ellipsometry. Furthermore, one has to consider that host materials can be birefringent, if they form so-called anisotropic molecular glasses (Yokoyama, 2011) (Ediger et al., 2019)), as discussed further below. During analysis

the n and κ values of the neat host films were used; however, they might change slightly in host-emitter films, which may be a possible source of error in the fitted Θ values. Nevertheless, the overall errors for the determination of the TDM orientation parameters shown in Figs. S2 and S3 are relatively small and do not affect the overall trend seen in the different host materials and the conclusions drawn from it in the main manuscript.

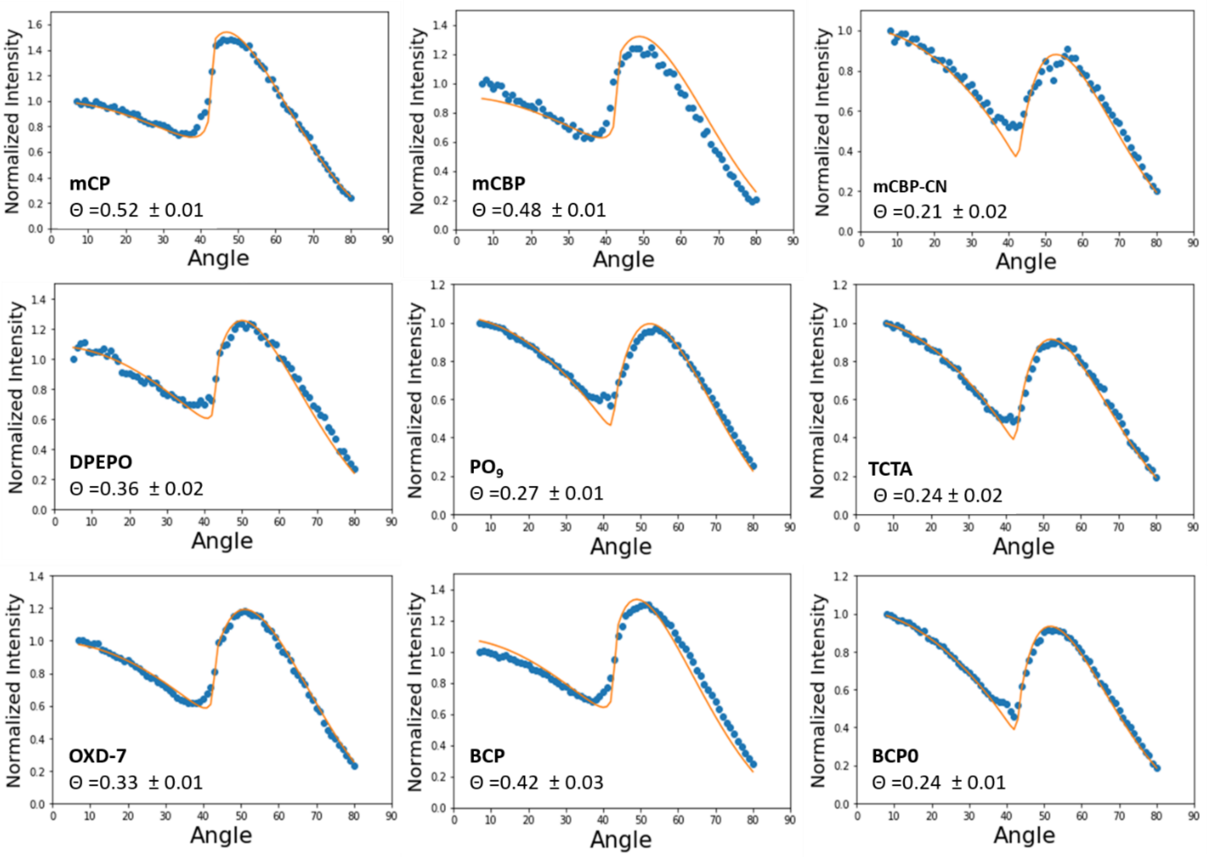


*Figure S2: Measured (blue dots) and fitted (orange lines) ADPL spectra of DMAC-TRZ in different hosts. The TDM orientation parameter is given in each case, which is obtained by numerical fitting.*

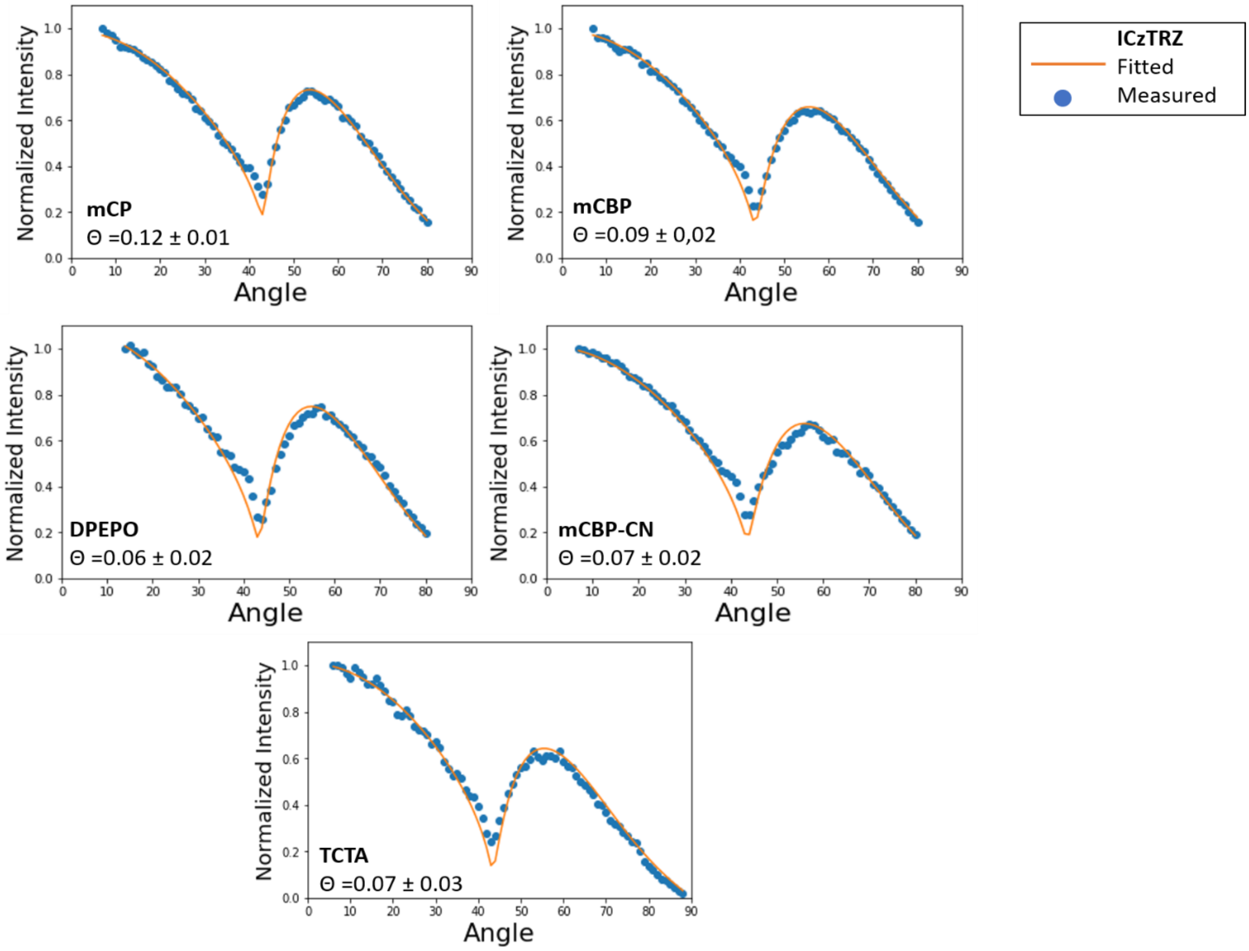


*Figure S3: Measured (blue dots) and fitted (orange lines) ADPL spectra of ICzTRZ in different hosts. The TDM orientation parameter is given in each case, which is obtained by numerical fitting.*

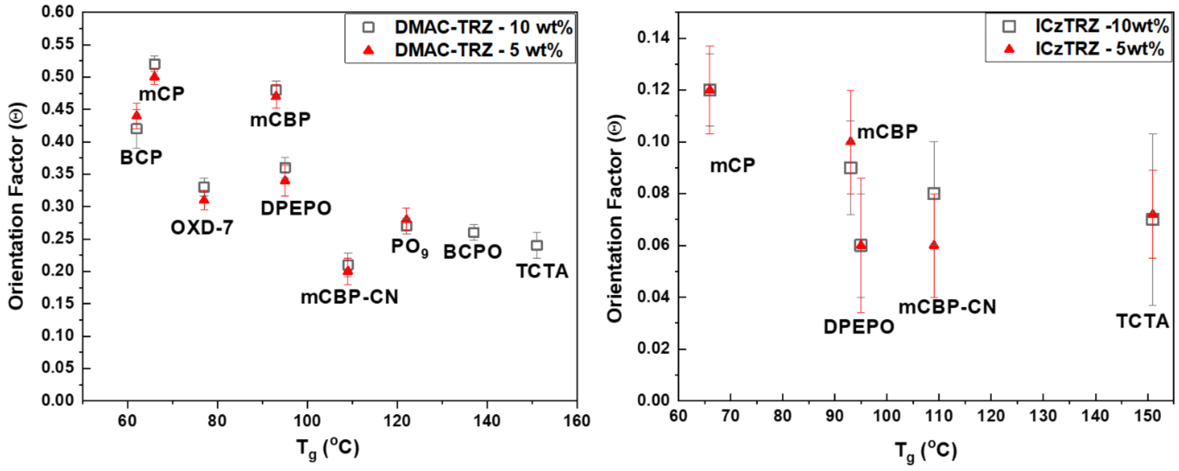


*Figure S4: Orientation factors for DMAC-TRZ (left) and ICzTRZ (right) with 5 and 10 wt% in different hosts. The values are in good agreement within the error range of each other for both concentrations. The 10% values are used in the manuscript.*

## 2. Refractive indices of host materials

The optical constants of the host materials were investigated by variable angle spectroscopic ellipsometry with a Sentech SE850 ellipsometer. After the measurement the obtained Ψ and Δ values were fitted with the Software Spectra Ray 3 to determine the refractive index as well as the extinction coefficient dispersion curves. Potential birefringence is considered by performing measurements and modelling at multiple angles.

The resulting data are shown in Fig. S5. The refractive index dispersion is mostly relevant for optical simulation in the spectral range of emission of the TADF emitters (i.e. approximately 400-650 nm), whereas the extinction coefficient is additionally needed to discuss excitation conditions of the used incident laser at 325 nm.

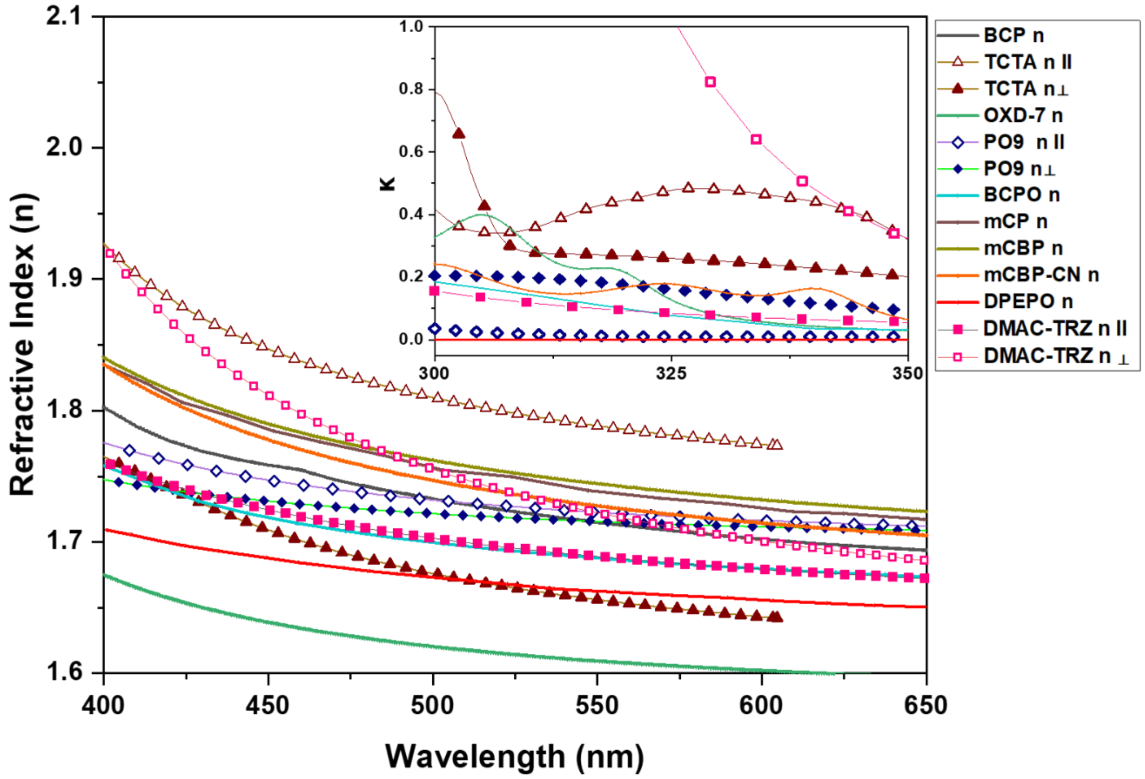


*Figure S5: Refractive index (n) and extinction coefficient (κ) in the inset. TCTA and $PO_9$ have two components on n-κ, which indicates birefringence. The same is true for a neat film of the emitter DMAC-TRZ, which is shown for completeness as well.*

It is worth noting that in most of the guest-host system energy transfer takes place between host and emitter when the substrate is irradiated with the 325nm laser; however, DPEPO is an exception, because it does not show measurable absorption above 300nm. Due to direct excitation of the emitter it is possible in this case to see the effect of photo-selection. One can

therefore not completely rule out that only certain TDM orientations are excited in the configuration of ADPL. This is a topic of ongoing research to investigate this effect.

**3. TD-DFT of emitter molecules**

At first the structure of emitter molecules was optimized in vacuum employing density functional theory (DFT) with the PBE0 functional (Adamo and Barone, 1999) and the 6-31G** basis set (Pople et al., 1976). This level of theory was chosen as it is able to reproduce the dihedral between the donor and acceptor units of ICzTRZ published elsewhere (Zhang et al., manuscript submitted) more accurately than other functionals (e.g. B3LYP). After that, time dependent (TD) calculations were performed using the Tamm-Dancoff approximation (TDA) (Moral et. al., 2015) in order to determine the natural transition orbitals and the transition dipole moments that are shown in the main publication. These orbitals and the TDM vectors refer to the excitation with the lowest energy and also exhibit the largest oscillator strength compared to all the transitions. All calculations were carried out with Jaguar (Bochevarov et al., 2013) as part of the Maestro Software Package of Schrödinger version 2019-4 (Schrödinger, 2019) and are in good agreement with previous studies (Lin et al., 2016; Zhang et al., manuscript submitted)

**4. Permanent dipole moments (PDMs) of host molecules**

In order to optimize the three-dimensional structures of the host molecules we first conducted a conformational search of the molecules in vacuum employing the molecular dynamics library MacroModel (Schrödinger) and the OPLS3 force field (Harder et al., 2016). After that we performed a density functional theory (DFT) geometry optimization for all relevant conformations with the a-posteriori functional B3LYP-D3 and the basis 6-31G** in the DFT code Jaguar (Bochevarov et al., 2013) . Both programs are part of the Maestro Software Package of Schrödinger version 2019-4 (Schrödinger, 2019). Some of the hosts in this study exhibit multiple stable conformations with similar gas phase energy but notable differences in molecular geometry and PDM, as already reported for OXD-7 (Emelyanova et al., 2013). For example, mCBP shows many different conformations in the gas phase, due to the possible rotation of the two carbazole groups. More importantly, the molecule is additionally able to rotate around the central single bond between the phenyl rings as shown in Figure S5. We identified two possible configurations of this dihedral angle rotations leading to PDMs of 0.8 D and 2.1 D for mCBP, respectively.

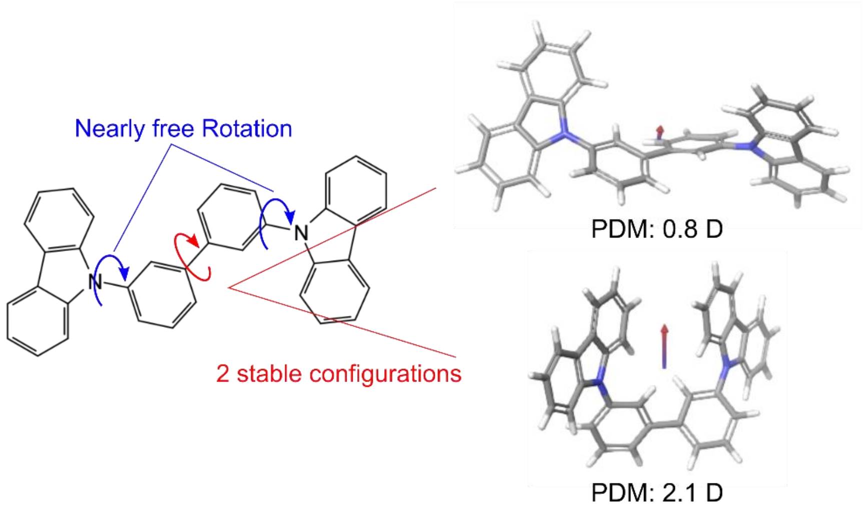


*Figure S6: The 3 single bonds in mCBP allow for a large number of conformations with nearly equal energy. While the rotation of the carbazole is nearly free (blue arrow) only two configurations for the rotation around the central single bond are stable (red), defining the possible PDMs of the molecule.*

In order to estimate the average permanent dipole moment in a film, we calculated the Boltzmann population with the gas phase energy of the different components considering all predicted stable conformations and weighted them accordingly. Figure S7 shows an overview of the predicted structures for the other 7 polar host materials. (Not shown is TCTA, where the literature does not report any significant PDM.) The values for the permanent dipole moments are in good agreement with available published data (Emelyanova et al., 2013; Noguchi et al., 2019), except for DPEPO. In this case values ranging from 2.2 D (Ahn et al., 2018) to 8.1 D (Northey et al., 2017) have been reported, already indicating its conformational flexibility. In previous work, the performance of TADF materials in DPEPO was related to a reorganization of the molecule in thin films (Deng et al., 2019), stating that even in the solid state DPEPO can geometrically relax and adjust its PDM to stabilize the excited state of a TADF emitter. We therefore conclude that for DPEPO the gas phase calculation and a Boltzmann population of the available states in the gas phase does no longer govern the situation in the film. Instead, preliminary calculations reflecting the actual film morphology indicate that there is a broad distribution of PDMs in DPEPO films leading to an average value in the range of 5 to 6 D. Thus, we use a value of about 5.5 D for the calculations of the degree of PDM alignment performed in the main manuscript.

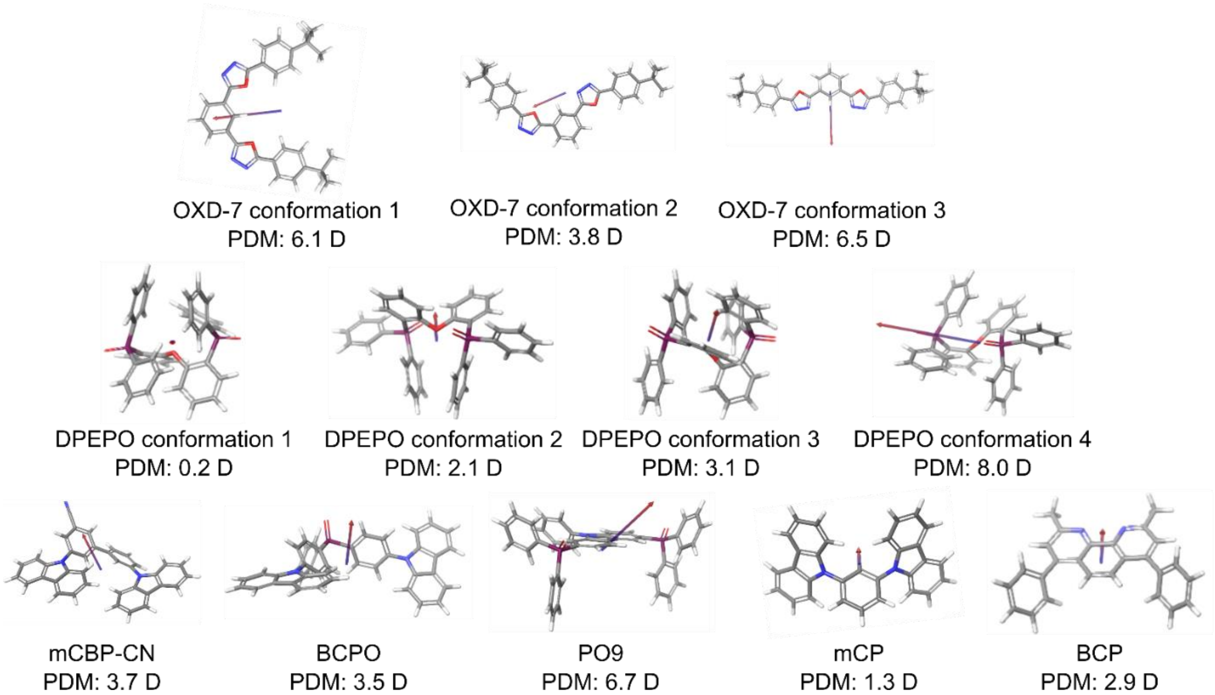


*Figure S7: Overview of the optimized 3D gas-phase structures and the PDMs of the polar hosts, used in this study. In case when there are multiple conformers with different PDM for the same molecule, one exemplary configuration for each PDM is shown. From the Boltzmann population of all the calculated conformations we expect an average PDM of 5.5 D for OXD-7 and 2.1 D for DPEPO; however, as discussed in the text the latter value is not realistic for thin films of DPEPO.*

## 5. Impedance spectroscopy and permanent dipole orientation of host materials

We used impedance spectroscopy on a simplified device structure as shown in Figure S8 in order to study the giant surface potential (GSP) and the orientation of the permanent dipole moments of the host molecules. If polar molecules show an orientation of their PDMs in a way that a negative charge density is formed at the interface to a non-polar hole transport layer (HTL), holes are injected into the HTL even at voltages below the built-in potential $V_{bi}$ (Berleb et al., 2000). This behavior leads to a characteristic step in the capacitance-voltage curve as it is visible for BPCO, PO9, DPEPO and mCBP-CN in Figure S8. For the other polar materials, we refer to previous studies as summarized, e.g., by Noguchi et al (Noguchi et al., 2019).

The onset voltage of the step in the C-V-measurement is usually referred to as the transition-voltage $V_t$ and the value of the capacitance at the plateau is typically termed $C_{ETL}$ as only the ETL is acting like a dielectric in this regime (Nowy et al., 2010). From these values the GSP of the polar ETL material can be calculated according to

$$GSP = \frac{C_{ETL}}{\varepsilon_r \varepsilon_0 A} \cdot (V_t - V_{bi})$$

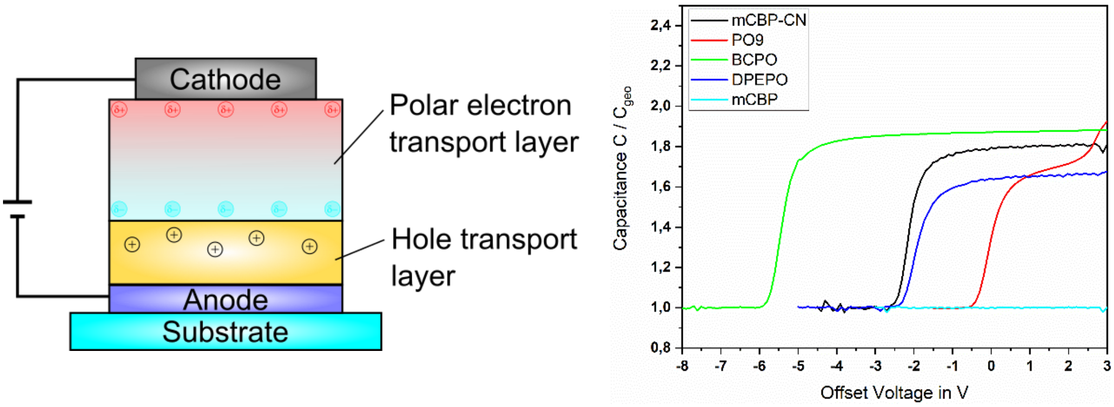


*Figure S8: (Left) Schematic sketch of the device structure used for impedance spectroscopy measurements. The anode consists of 150 nm pre-structured ITO with PEDOT:PSS spin-coated on top for hole injection. The active device layers are 70 nm of NPB as a non-polar HTL and typically 70 nm of the polar material under investigation. A cathode with 15 nm Calcium and 100 nm of Aluminum are evaporated as top contact. (Right) Exemplary capacitance voltage characteristics of the device structure with mCBP-CN, BCPO, PO9, DPEPO and mCBP. The missing transition in the C-V curve of mCBP indicates that this material does not show any GSP in impedance spectroscopy.*

with the dielectric constant of the ETL material $\varepsilon_r$, the permittivity of vacuum $\varepsilon_0$ and the active device area A (Brütting et al., 2001). In order to obtain information about the average

orientation of the permanent dipole moments, we calculate the fraction of oriented dipoles Λ. This order parameter is given by dividing the measured GSP through the maximum possible $GSP_{max}$ for a film of perfectly vertically aligned dipoles

$$\Lambda = \frac{GSP}{GSP_{max}}, \text{with } GSP_{max} = \frac{1}{\varepsilon_r \varepsilon_0} \cdot PDM \cdot \frac{\rho \cdot N_A}{M},$$

where $\rho, N_A$ and $M$ describe the density of the ETL, Avogadro's number and the molar mass of the investigated material, respectively (Morgenstern et al., 2018)(Jäger et al., 2016). We obtained the density of the different compounds, where no literature values for amorphous films were available, from a molecular dynamics simulation with Desmond (Bowers et al., 2006) and the OPLS3 force field (Harder et al., 2016) within the Schrödinger Materials Science Suite. The density values (in g/cm$^3$) are 1.19 for DPEPO, 1.15 for mCP, 1.13 for mCBP-CN, 1.13 for OXD-7 (Emelyanova et al., 2013), 1.11 for BCP, 1.15 for BCPO and 1.16 for PO9. For the calculation of the GSP we estimated the dielectric constant to be roughly 3.0. Please note that this assumption does not affect the calculated Λ parameters because both the expressions for the GSP and $GSP_{max}$ contain this value. Only in cases when the GSP value has been determined by other methods, e.g. via Kelvin probe, this value contributes to the error of the order parameter.

## 6. Device structure and characterisation

A study of potential TADF OLEDs was conducted using DMAC-TRZ as the emitter material with one of the hosts discussed in the manuscript. mCBP-CN was selected as host material since the orientation measurements indicate horizontal alignment of DMAC-TRZ in this host ($\theta$= 0.21) and mCBP-CN is known for its bipolar charge transport properties.

The fabricated device structure consisted of ITO (90 nm) with a spin-coated layer of HIL1.3 as anode. HIL1.3 is a PEDOT:PSS-type of conducting polymer used to enhance the work function of ITO for improved hole injection. This was followed by thermal vacuum evaporation of NPB (25 nm) as hole transport layer (HTL). Then, a layer of mCBP (10 nm) was deposited acting as an electron blocking layer before the deposition of the emitter-host system consisting of 10% DMAC-TRZ in mCBP-CN (10 nm). For the electron transport layer (ETL), a layer of neat T2T (10 nm) has been deposited followed by another layer of T2T (40 nm) doped with LiQ (30%) to enhance the electron injection and transport properties. To complete the stack, Aluminium (100 nm) was deposited as the cathode (Fig. S9).

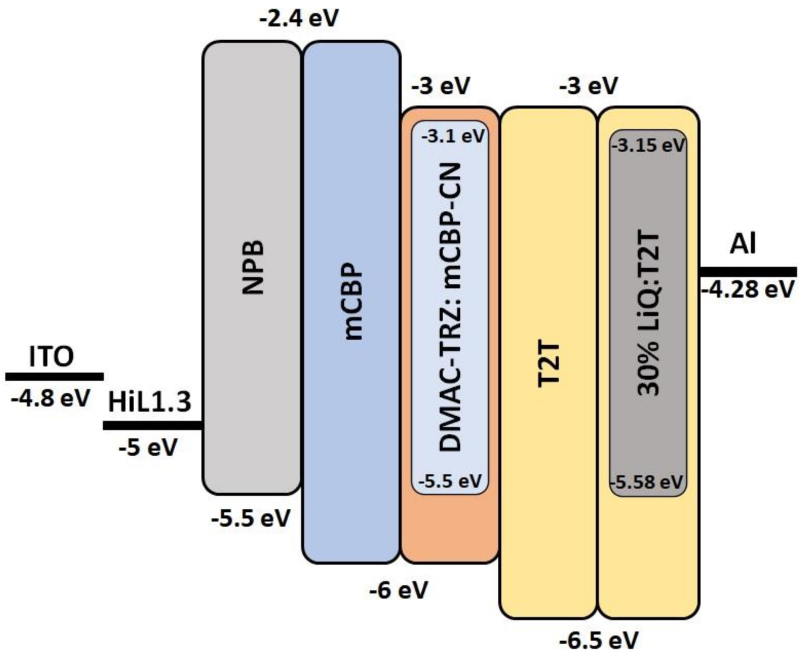


*Figure S9: Schematic of the fabricated device stack. Energy levels (work functions for the anode with hole-injection layer and the cathode, HOMO/LUMO levels for the organic layers and the emitter) are indicated.*

Current-voltage-luminance characteristics were measured with a source-measure unit (Keithley 2600) and a calibrated spot photometer (JETI Spectro-Radiometer). Using a Lambertian radiation pattern the obtained luminance was converted to external quantum efficiency values.

The OLED shows an $EQE_{max}$ of 12.8 % at 53 cd/m$^2$ with a low turn on voltage of 2.4 volts (Figure S10 a&b). The electroluminescence (EL) spectrum in Figure S10c is used to calculate the CIE coordinates of (0.23, 0.45) for the emitted light.

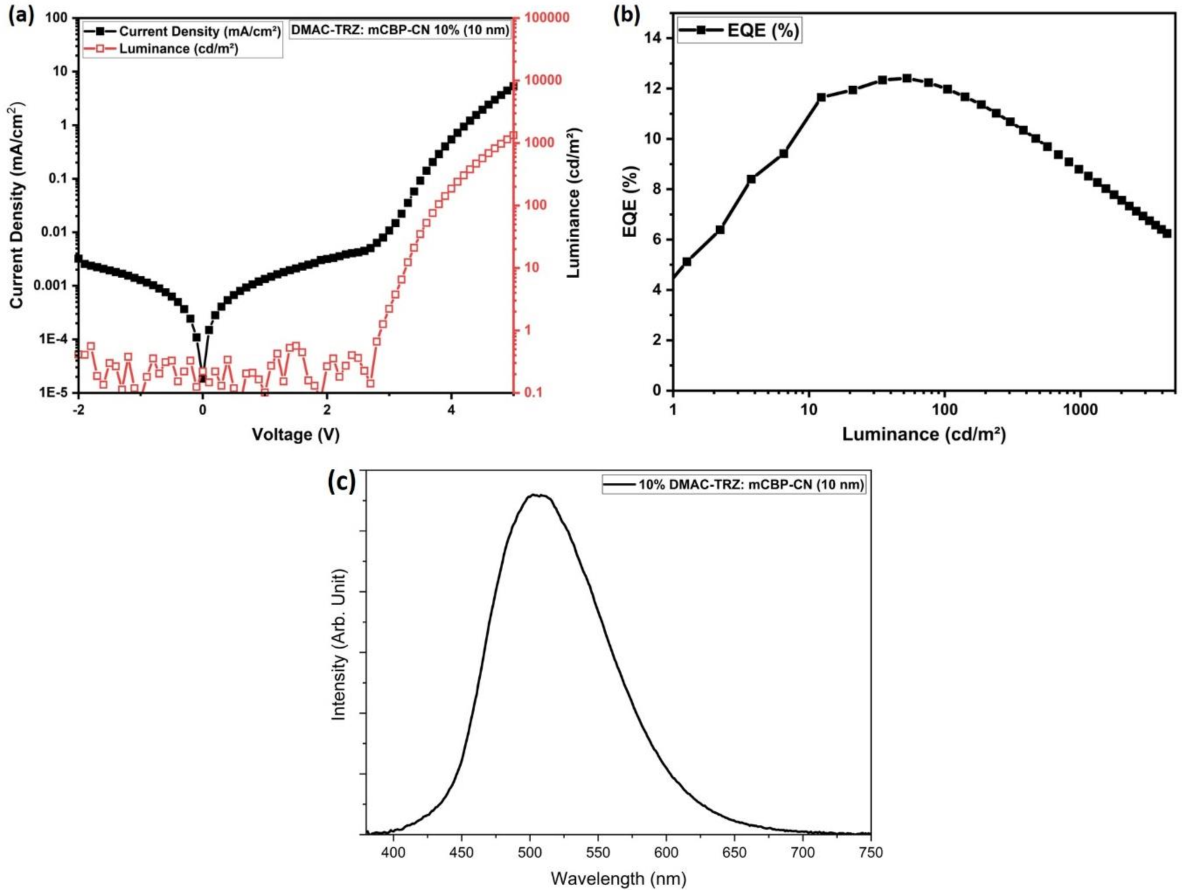


*Figure S10: Device characteristics of the fabricated OLED shown in Fig. S9. (a) J-V-L curve, (b) EQE vs luminance, and (c) electroluminescence spectrum.*

The simulation of light outcoupling from OLEDs, shown in the main manuscript, has been performed with a dipole model as described in detail in Ref. (Schmidt et al. 2017). Therein the charge carrier balance for the device is taken to be 1, i.e. equal injection of holes and electrons and unity recombination in the device are assumed. This would result in an EQE-vs.-luminance plot, where the EQE is constant for low light intensity followed by a roll-off above a critical luminance.

However, as visible in figure S9(b), the EQE curve resembles an inverted parabola. We observe a significant rise in EQE followed by a decrease past the max. EQE with increasing luminance. This can be attributed to a non-ideal charge carrier balance significantly less than unity, which apparently improves with increasing current until the competing roll-off takes over. We assume that this imbalance of charge carriers is the main reason for the discrepancy between the simulated light outcoupling and the actually measured max. EQE. However, it needs further

analysis and modification of the device stack, for example variation of the thickness of HTL and ETL, to get a better performance. We note that in a separate publication (Zhang et al. manuscript submitted) we obtained almost quantitative agreement between experiment and simulation for OLEDs based on ICzTRZ as emitter.

### 7. Differential scanning calorimetry on host materials

Differential scanning calorimetry of the hosts mCBP-CN, DPEPO, mCBP and mCP and as well as emitters DMAC-TRZ and ICzTRZ was performed with a Mettler DSC3+ in pierced Al pans under nitrogen flow. The glass transition temperature ($T_g$) was determined as the inflection point of the step in the second heating scan at 10 K/min (Fig. S11). Every compound has been subjected to 4 heating and cooling cycles. The heatings were made at different rates (10 K/min, 10 K/min, 20 K/min and 40 K/min) to get reproducible data while the coolings were made with a high rate of 100 K/min in order to induce an amorphous state of the compounds. The heating scans of DPEPO are shown in Fig. S11a (for clearance the coolings are excluded and the focus is on the $T_g$). The first heating doesn't show any glass transition because the compound is fully crystalline with a melting point at 281°C. From the second heating on, due to the fast cooling rate, it is possible to see the $T_g$ value at 93°C (and recrystallization around 180°C). In particular, by increasing the heating speed, the glass transition gets more pronounced and shifts to higher temperatures due to a delay in the instrument's response. For this reason, the value of $T_g$ is taken from the second heating run. Fig S11b shows DSC heating scans of mCBP-CN. In the first run there is only the melting point at 256 °C and no recrystallization is observed. Upon fast cooling (100K/min) 2$^{nd}$, 3$^{rd}$ and 4$^{th}$ heatings show $T_g$ at 113°C. mCP shows a very similar phase behaviour with a melting at 182°C and a low glass transition at 65°C. The phase behaviour of mCBP with a $T_g$ of 92°C is more complex. In all scans starting from the 2$^{nd}$, heating recrystallization is observed at about 150°C before melting at 269°C. For the carbazole-based molecules it is possible to see a clear increase of $T_g$ going from the smallest molecule (mCP) to the bulkiest one (mCBP-CN). mCBP shows an intermediate $T_g$, similar to the one of DPEPO.

DMAC-TRZ shows a glass transition at 93 °C and a melting point at 234 °C. ICzTRZ, due to the higher molecular weight, shows a $T_g$ at 253 °C and a melting point at 418 °C followed by a recrystallization at 423°C and another melting point at 447 °C (Fig. S12).

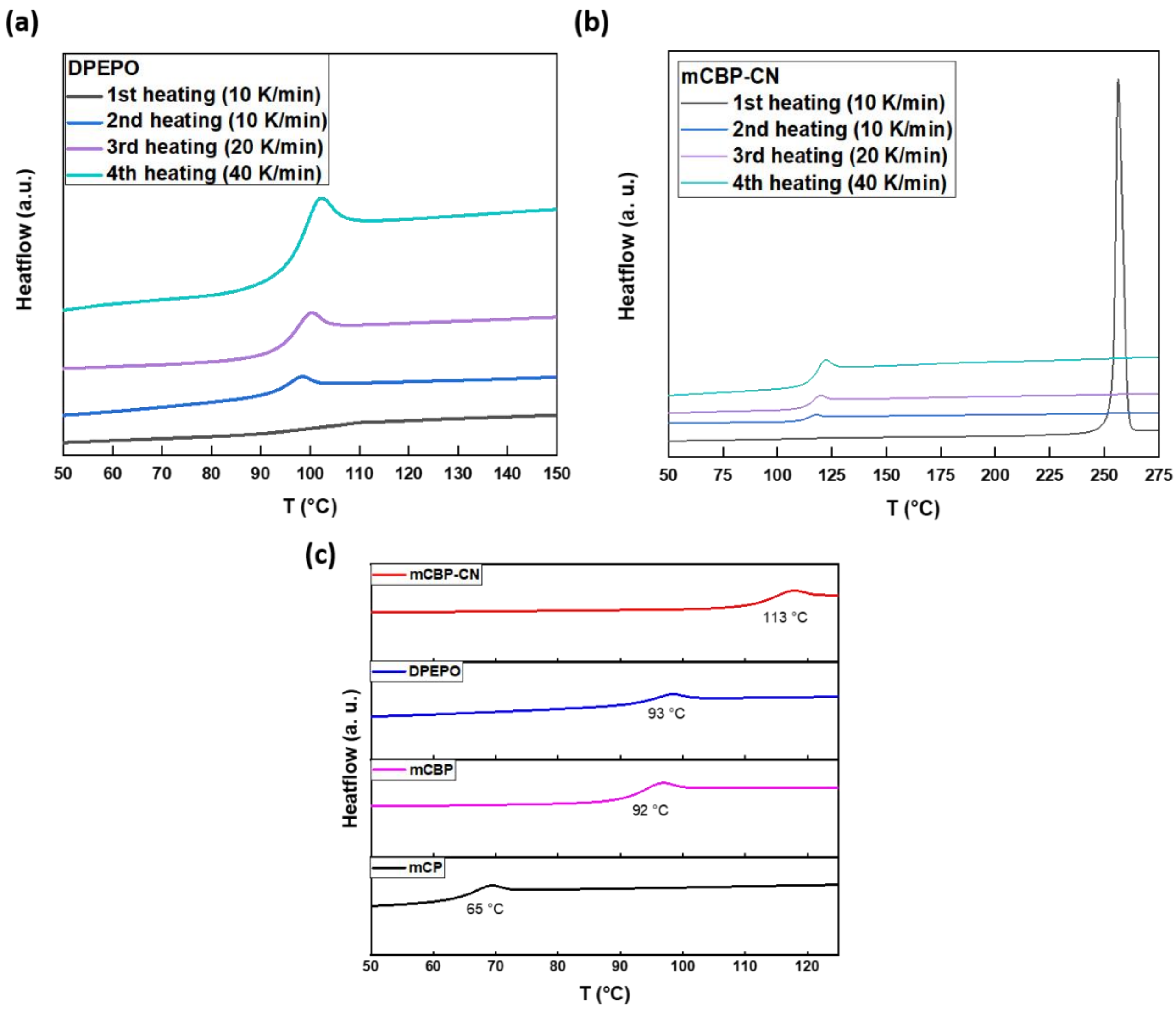


*Fig. S11: Heating scans of (a) DPEPO and (b) mCBP-CN with focus on $T_g$. (c) Glass transition temperatures of the hosts mCBP-CN, DPEPO, mCBP and mCP are derived from the second heating scan as described in the text.*

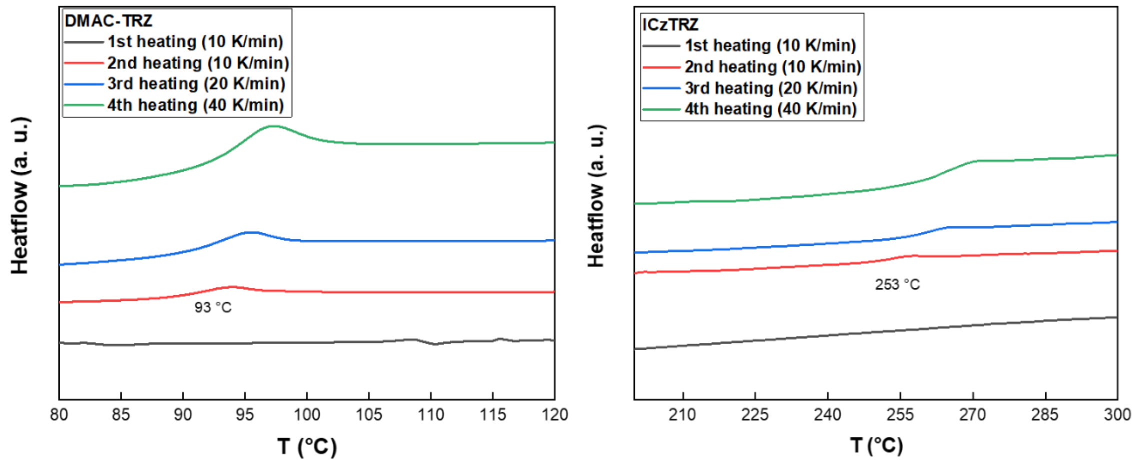


*Fig. S12: Heating scans of DMAC-TRZ (left) and ICzTRZ (right) with focus on $T_g$. The glass transition temperatures are derived from the second heating scan are as indicated.*

## 8. References


Adamo, C., and Barone, V. (1999). Toward reliable density functional methods without adjustable parameters: The PBE0 model. *J. Chem. Phys.* 110, 6158–6170. doi:10.1063/1.478522.

Ahn, D. H., Moon, J. S., Kim, S. W., Lee, S. Y., Karthik, D., Lee, J. Y., et al. (2018). Effect of various host characteristics on blue thermally activated delayed fluorescent devices. *Org. Electron.* 59, 39–44. doi:10.1016/j.orgel.2018.04.034.

Berleb, S., Brütting, W., and Paasch, G. (2000). Interfacial charges and electric field distribution in organic hetero-layer light-emitting devices. *Org. Electron.* 1, 41–47. doi:10.1016/S1566-1199(00)00007-0.

Berleb, S., Brütting, W., and Paasch, G. (2001). Interfacial charges in organic hetero-layer light emitting diodes probed by capacitance-voltage measurements. *Synth. Met.* 122, 37–39. doi:10.1016/S0379-6779(00)01356-4.

Bochevarov, A. D., Harder, E., Hughes, T. F., Greenwood, J. R., Braden, D. A., Philipp, D. M., et al. (2013). Jaguar: A high-performance quantum chemistry software program with strengths in life and materials sciences. *Int. J. Quantum Chem.* 113, 2110–2142. doi:10.1002/qua.24481.

Bowers, K. J., Chow, E., Xu, H., Dror, R. O., Eastwood, M. P., Gregersen, B. A., et al. (2006). Scalable algorithms for molecular dynamics simulations on commodity clusters. *Proc. 2006 ACM/IEEE Conf. Supercomput. SC'06*. doi:10.1145/1188455.1188544.

Deng, C., Zhang, L., Wang, D., Tsuboi, T., and Zhang, Q. (2019). Exciton- and Polaron-Induced Reversible Dipole Reorientation in Amorphous Organic Semiconductor Films. *Adv. Opt. Mater.* 7, 1–7. doi:10.1002/adom.201801644.

Ediger, M. D., De Pablo, J., and Yu, L. (2019). Anisotropic Vapor-Deposited Glasses: Hybrid Organic Solids. *Acc. Chem. Res.* 52, 407–414. doi:10.1021/acs.accounts.8b00513.

Emelyanova, S., Chashchikhin, V., and Bagaturyants, A. (2013). Atomistic multiscale simulation of the structure and properties of an amorphous OXD-7 layer. *Chem. Phys. Lett.* 590, 101–105. doi:10.1016/j.cplett.2013.10.047.

Frischeisen, J., Yokoyama, D., Adachi, C., and Brütting, W. (2010). Determination of molecular dipole orientation in doped fluorescent organic thin films by

photoluminescence measurements. *Appl. Phys. Lett.* 96, 1–4. doi:10.1063/1.3309705.

Harder, E., Damm, W., Maple, J., Wu, C., Reboul, M., Xiang, J. Y., et al. (2016). OPLS3: A Force Field Providing Broad Coverage of Drug-like Small Molecules and Proteins. *J. Chem. Theory Comput.* 12, 281–296. doi:10.1021/acs.jctc.5b00864.

Jäger, L., Schmidt, T. D., and Brütting, W. (2016). Manipulation and control of the interfacial polarization in organic light-emitting diodes by dipolar doping. *AIP Adv.* 6, 1–7. doi:10.1063/1.4963796.

Lin, T. A., Chatterjee, T., Tsai, W. L., Lee, W. K., Wu, M. J., Jiao, M., et al. (2016). Sky-Blue Organic Light Emitting Diode with 37% External Quantum Efficiency Using Thermally Activated Delayed Fluorescence from Spiroacridine-Triazine Hybrid. *Adv. Mater.* 28, 6976–6983. doi:10.1002/adma.201601675.

Morgenstern, T., Schmid, M., Hofmann, A., Bierling, M., Jäger, L., and Brütting, W. (2018). Correlating Optical and Electrical Dipole Moments To Pinpoint Phosphorescent Dye Alignment in Organic Light-Emitting Diodes. *ACS Appl. Mater. Interfaces* 10, 31541–31551. doi:10.1021/acsami.8b08963.

Noguchi, Y., Brütting, W., and Ishii, H. (2019). Spontaneous orientation polarization in organic light-emitting diodes. *Jpn. J. Appl. Phys.* 58. doi:10.7567/1347-4065/ab0de8.

Northey, T., Stacey, J., and Penfold, T. J. (2017). The role of solid state solvation on the charge transfer state of a thermally activated delayed fluorescence emitter. *J. Mater. Chem. C* 5, 11001–11009. doi:10.1039/c7tc04099g.

Nowy, S., Ren, W., Elschner, A., Lövenich, W., and Brütting, W. (2010). Impedance spectroscopy as a probe for the degradation of organic light-emitting diodes. *J. Appl. Phys.* 107, 54501. doi:10.1063/1.3294642.

Pople, J. A., Binkley, J. S., and Seeger, R. (1976). Theoretical models incorporating electron correlation. *Int. J. Quantum Chem.* 10, 1–19. doi:10.1002/qua.560100802.

Schmidt, T. D., Lampe, T., Daniel Sylvinson, M. R., Djurovich, P. I., Thompson, M. E., and Brütting, W. (2017). Emitter Orientation as a Key Parameter in Organic Light-Emitting Diodes. *Phys. Rev. Appl.* 8, 1–28. doi:10.1103/PhysRevApplied.8.037001.

Schrödinger Schrödinger Release 2019-4, Macromodel. Available at: https://www.schrodinger.com/macromodel.

Schrödinger (2019). Schrödinger Release 2019-4: Maestro.

Yokoyama, D. (2011). Molecular orientation in small-molecule organic light-emitting diodes. *J. Mater. Chem.* 21, 19187–19202. doi:10.1039/c1jm13417e.

Z.Zhang, E Crovini, P. L. D Santos, B. Naqvi, P. Sahay, D. Cordes, A. Slawin, W. Bruetting, I. D. W. Samuel, S. B. and E. Z. C. (2020). Efficient Sky-Blue Organic Light-emitting Diodes Using a Highly Horizontally Oriented Thermally Activated Delayed Fluorescence Emitter. *Manuscr. Submitt.*
